# Allelic variation contributes to bacterial host specificity

**DOI:** 10.1038/ncomms9754

**Published:** 2015-10-30

**Authors:** Min Yue, Xiangan Han, Leon De Masi, Chunhong Zhu, Xun Ma, Junjie Zhang, Renwei Wu, Robert Schmieder, Radhey S. Kaushik, George P. Fraser, Shaohua Zhao, Patrick F. McDermott, François-Xavier Weill, Jacques G. Mainil, Cesar Arze, W. Florian Fricke, Robert A. Edwards, Dustin Brisson, Nancy R. Zhang, Shelley C. Rankin, Dieter M. Schifferli

**Affiliations:** 1 Department of Pathobiology, University of Pennsylvania School of Veterinary Medicine, 3800 Spruce St, Philadelphia, Pennsylvania 19104, USA; 2 Department of Computer Science, College of Sciences, San Diego State University, 5500 Campanile Drive, PS 106, San Diego, California 92182, USA; 3 Department of Veterinary and Biomedical Sciences, South Dakota State University, Brookings, South Dakota 57007, USA; 4 Department of Biology and Microbiology, Box-2140D, South Dakota State University, Brookings, South Dakota 57007, USA; 5 Pennsylvania Department of Health, Bureau of Laboratories, 110 Pickering Way, Exton, Pennsylvania 19341, USA; 6 Division of Animal and Food Microbiology, Center for Veterinary Medicine, US FDA, Office of Research, 8401 Muirkirk Road, Laurel, Maryland 20708, USA; 7 Institut Pasteur, Unité des Bactéries Pathogènes Entériques, 28 rue du Docteur Roux, 75724 Paris Cedex 15, France; 8 Bacteriology, Department of Infectious Diseases, Faculty of Veterinary Medicine and Institute for Fundamental and Applied Research in Animal Health (FARAH), Campus du Sart Tilman, Bât. B43a, University of Liège, 4000 Liège, Belgium; 9 Institute for Genome Sciences, Department for Microbiology and Immunology, University of Maryland School of Medicine, Baltimore, 801W Baltimore St, Maryland 21201, USA; 10 Mathematics and Computer Science Division, Argonne National Laboratory, 9700S. Cass Avenue, Argonne, Illinois 60439, USA; 11 Department of Biology, School of Art and Science, University of Pennsylvania, 209 Leidy Laboratories, 433S. University Avenue, Philadelphia, Pennsylvania 19104, USA; 12 Department of Statistics, Wharton School, University of Pennsylvania, 3730 Walnut Street, Philadelphia, Pennsylvania 19104, USA

## Abstract

Understanding the molecular parameters that regulate cross-species transmission and host adaptation of potential pathogens is crucial to control emerging infectious disease. Although microbial pathotype diversity is conventionally associated with gene gain or loss, the role of pathoadaptive nonsynonymous single-nucleotide polymorphisms (nsSNPs) has not been systematically evaluated. Here, our genome-wide analysis of core genes within *Salmonella enterica* serovar Typhimurium genomes reveals a high degree of allelic variation in surface-exposed molecules, including adhesins that promote host colonization. Subsequent multinomial logistic regression, MultiPhen and Random Forest analyses of known/suspected adhesins from 580 independent Typhimurium isolates identifies distinct host-specific nsSNP signatures. Moreover, population and functional analyses of host-associated nsSNPs for FimH, the type 1 fimbrial adhesin, highlights the role of key allelic residues in host-specific adherence *in vitro*. Together, our data provide the first concrete evidence that functional differences between allelic variants of bacterial proteins likely contribute to pathoadaption to diverse hosts.

## Abstract (web summary)

One of the key aspects for controlling infectious diseases is understanding how pathogens cross host species. Here the authors conduct a genome-wide analysis of *Salmonella* and show a high degree of variation, enabling host-adapted colonization among *Salmonella* intestinal and systemic serovars.

## Graphical abstract

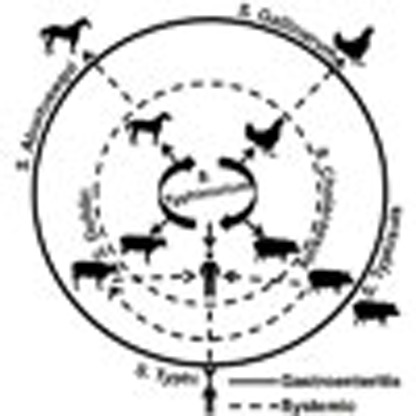

Evolutionary events that modulate interactions between a pathogen and its host have a critical impact on interspecies transmission and adaptation, and thus on host range and pathogenesis^1^. For example, although most avian influenza viruses remain restricted to birds, some, such as H5N1 and H7N9, cause serious infections in humans^2^. The ability of influenza viruses to cross host barriers is determined by the amino-acid sequence of its haemagglutinin protein, a lectin that recognizes sialylated glycan receptors on the apical surface of host cells. Even small changes of a few amino acids in the haemagglutinin protein are sufficient to convert receptor specificity from avian to human^1^,^2^. The exact mechanisms for host tropism and adaptation of bacterial pathogens such as *Salmonella* remain elusive, and what is known has been primarily revealed by either gene-centric functional investigations^3^,^4^,^5^ or by genomic studies^6^,^7^. Moreover, the biological relevance of most host-specific associations identified in genome-wide studies remains untested^8^,^9^,^10^.

The ∼1,500 closely related but distinct *Salmonella enterica* subsp. *enterica* serovars, determined by lipopolysaccharides and flagellar antigens, can be divided into three groups based on epidemiological host prevalence. Many *S. enterica* serovars such as *S. enterica* serovar Typhimurium (*S.* Typhimurium) are restricted to the intestine, and cause limited clinical or subclinical enteric infections in a variety of unrelated hosts. In contrast, several *S. enterica* serovars that are particularly well adapted to their host, including Typhi in humans and Gallinarum in poultry, are more invasive, and result in a systemic infection that can be lethal if not treated promptly with antibiotics. However, epidemiological evidence supports various levels of host adaptation even among strains of broad host range serovars. For example, whereas most strains of *S.* Typhimurium cause a typhoid-like disease in susceptible mice, particular phage types such as DT2 or DT99 can cause systemic infections in pigeons^11^ and the multi-locus sequence type ST313 causes systemic infection in humans and chickens^12^,^13^. Thus, it appears that both inter- and intra-serovar variation have a role in host range and disease severity. Here we undertook a genome-wide search to identify genomic sequences that contribute to host adaptation and surprisingly found that allelic variants of shared surface adhesion molecules correlated most strongly with host specificity. Most importantly, functional analysis of identified variants of the FimH adhesin confirmed their biological relevance in modulating host-specific binding that can contribute to host-adaptation and ultimately to the *Salmonella* strain pathotype.

## Results

### Detection of genome-host associations in *S.* Typhimurium

**Figure 1:**
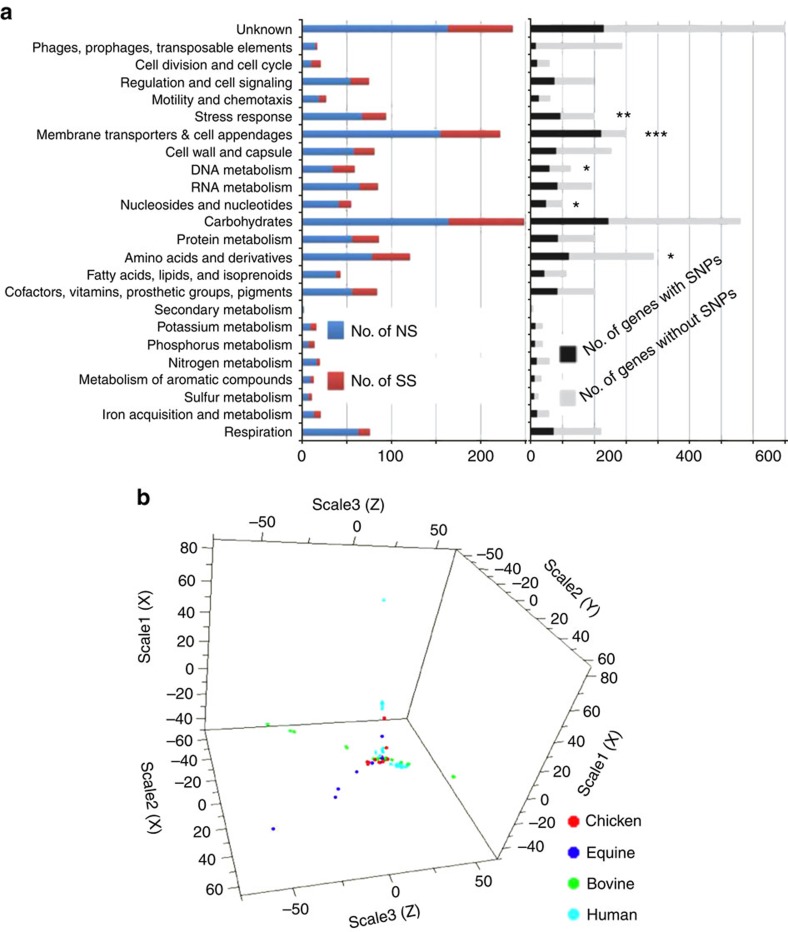
Comparative analysis and host origin association for *S.* Typhimurium genomes. (**a**) Functional distribution of core genes in 12 isolates; at left total numbers of SNPs with proportions of nonsynonymous substitutions (NS, blue) and synonymous substitutions (SS, red), and at right, the number of genes with (black) and without SNPs (grey). *P*-values for associations of sets of genes with SNPs and a given functional category (right-tailed Fisher exact test): *P<0.05; **P<0.01; ***P<0.001. (**b**) 3D scaling plot from a Random Forest proximity matrix of the SNPs from 15 adhesins (using the first 3 principal components) for human (light blue), bovine (green), equine (dark blue) and chicken (red) isolates; the analysis identified host-specific DNA signatures by separating subpopulations of isolates from the same host.

To determine what genomic changes contribute to host association in *Salmonella*, we focused on *S.* Typhimurium, a broad-host range serovar for which the molecular basis for host preferences remains essentially unknown. A comparative analysis of the 3,192 core genes from 12 available complete genomes of *S.* Typhimurium (Supplementary Fig. 1a) detected a relative and preferential accumulation of single-nucleotide polymorphisms (SNPs) among the annotated genes for membrane-associated surface and exported proteins (Fig. 1a). Most SNPs (69%) were nonsynonymous (nsSNPs). Noticeably, nsSNPs in the core genomes associated with specific hosts, as shown in a heat map of nsSNPs (Supplementary Fig. 1b), suggesting that host preferences of individual Typhimurium strains involves unique combinations of cell surface and exported allelic proteins. In contrast, no host-specific associations were identified in the 2,312 partially shared genes, which were frequently associated with loci carrying phage DNA (Supplementary Fig. 1c and Supplementary Table 1), the 1,207 unique genes, which were mostly mobile elements (Supplementary Fig. 1c,d), the 19 different plasmids (Supplementary Data 1), the few detected genomic rearrangements (Supplementary Fig. 2), or the shared pseudogenes (Supplementary Fig. 3). As the most distinctive genomic property of *S.* Typhimurium’s association to diverse hosts was its allelic variants of surface or exported proteins, we further investigated the potential role of a representative set of these proteins in host–pathogen interactions that may contribute to host adaptation.

### Distinct *S.* Typhimurium adhesin SNPs are host specific

Adhesive proteins or ligands on bacteria are likely to have an essential role in initiating host–pathogen interactions that contribute to host adaptation. Therefore, we undertook a population-scale analysis of 580 Typhimurium strains and focused on 12 known or predicted fimbrial adhesins and 3 outer membrane proteins suspected to have binding properties. Sequence data were collected from 198 available genomes (Supplementary Data 2) and from targeted sequencing of 382 independent isolates (Supplementary Data 3)^14^. We first investigated the degree of variability for the 15 genes, and found that *fimH* encoded the largest number of different alleles (Supplementary Table 2). Tajima’s *D* tests for non-neutral evolution suggested that all 15 genes underwent positive selection. SNPs from these 15 genes were further analysed for host association using Random Forest (RF), multinomial logistic regression and MultiPhen analyses (Supplementary Data 4–6)^15^,^16^. A total of 182 host-associated mutations were detected by at least one method, 82 by two methods and 32 by all three methods (Supplementary Fig. 5). Of the 32 consistently identified host-associated mutations, half were nsSNPs, two of which were related to the absence or presence of a full-length open-reading frame (Supplementary Table 3). Moreover, a 3D scaling plot from the RF analysis that separated subpopulations of isolates from the same host clearly revealed host-specific DNA signatures (Fig. 1b and Supplementary Fig. 4). Collectively, these *in silico* data strongly suggest that allelic variation in known or predicted bacterial adhesins of a broad-host range serovar is associated with host-specificity.

### A *S.* Typhimurium FimH residue shapes host-specific adhesion

**Figure 2:**
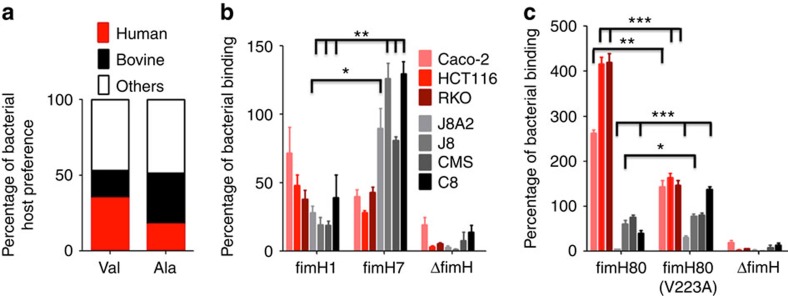
Residue 223 variation in FimH of *S.* Typhimurium and Typhi swaps host specificity. (**a**) Host origin distribution for 580 *S.* Typhimurium isolates that have either a valine (Val) or an alanine (Ala) at position 223 of FimH. (**b**) Binding to three human (in red) and four bovine (in black) intestinal epithelial cells of recombinant *E. coli* expressing *Salmonella* type 1 fimbriae with the FimH1 or FimH7 alleles that have valine or alanine at position 223, respectively. (**c**) The different binding properties of fimH80 with valine and engineered fimH80 with alaline at position 223 for three human and four bovine enterocytes. The data in **b** and **c** are expressed as mean percentages of bacterial binding relative to the difference between *fimH2* (100% binding for FimH of *S.* Typhimurium strain AJB3, not shown) and *ΔfimH* (0% binding) with±s.e.m. of three experiments. *P*-values were calculated by using a one-sided paired *t*-test: *P<0.05; **P<0.01; ***P<0.001.

The next goal was to evaluate whether a detected host-associated allelic variant effectively alters bacterial binding in a host-specific manner. Among the 15 genes studied in *S.* Typhimurium, we found that *fimH* encoded the greatest number of predicted alleles, with 17 identified variants, most of which were expressed in only one to three strains. However, two alleles were present in over 30 isolates. Although *fimH1* was a broad adaptive allele, it most frequently associated with human isolates, whereas the second most common allele, *fimH7*, was most frequently associated with isolates of bovine origin (Fig. 2a; *P*<0.0009, Fisher’s exact test). These two alleles differ by only one amino acid; with a valine for FimH1 and an alanine for FimH7 at position 223. To determine the potential biological function of this residue switch, we tested the binding property of recombinant *E. coli* expressing isogenic *S.* Typhimurium type 1 fimbriae with either one of the two allelic FimH proteins or no FimH as control. Using three human and four bovine intestinal epithelial cells, the affinity of *S.* Typhimurium FimH7 was greater than that of FimH1 for all bovine cells (Fig. 2b). Although FimH1 appeared to preferentially bind to some human cells, the difference was not statistically significant. Nevertheless, these results indicated that the alanine at position 223 in FimH7 has a role in bovine-specific adhesion. Like FimH1, all 48 available genome sequences of the human-restricted *S.* Typhi encode a valine at position 223. Thus, to determine whether this valine contributed to the preferential binding of *S.* Typhi FimH to human cells, we mutagenized the representative *fimH80* gene of *S.* Typhi by substituting its valine with an alanine. Bacteria expressing the mutated FimH80(V223A) allele demonstrated significantly decreased binding to all three human cells and increased binding to three of the four bovine cells investigated (Fig. 2c). Taken together, these results highlighted how unique nsSNPs in a bacterial adhesin from a broad-host range serovar contribute to bacterial–host interactions, and likely participate in host specificity.

### *fimH* variants coincide with distinct host-adapted *Salmonella*

**Figure 3:**
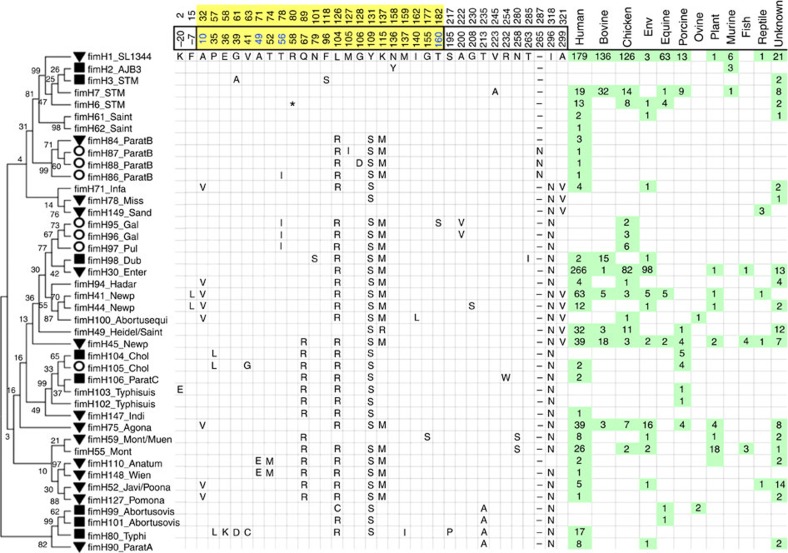
FimH protein sequence variants. Variant residue positions for the unprocessed (top line) and matured (second line) FimH proteins, with the FimH of strain SL1344 (FimH1) used as the comparative standard (third line). The star for Typhimurium *fimH6* is a stop codon. The signal peptide (22 residues), lectin (residues 1–173 of the mature protein, yellow background) and pilin domains (residues 177–315) are framed (second line). Substitutions and their corresponding positions are shown for each listed FimH. Variant residues that are predicted to participate in the mannose-binding pocket are highlighted in blue. At left, phylogenetic tree of the major *fimH* alleles (found in at least ten isolates per serovar; two, two and four isolates for serovars Typhisuis, Abortusovis and Abortusequi, respectively) based on nucleotide sequences and built by using the Maximum-likelihood method with a bootstrap value of 1,000. The mannose-binding properties of FimH are indicated as high binding (black square), low binding (black triangle) and nonbinding (white circle)^29^. On the right highlighted in green are the numbers of isolates studied for each listed allele and their origin (host, environment or unknown).

**Figure 4:**
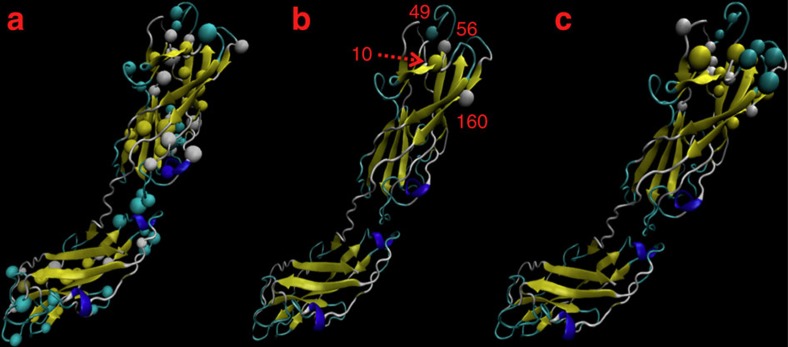
Predicted structure of *Salmonella* FimH1 (*S.* Typhimurium SL1344). The balls highlight the substituted residues. (**a**) All the amino-acid residues found to be substituted, as listed in Fig. 3; (**b**) all the substituted residues in the predicted binding pocket (Fig. 3); (**c**) all the residues predicted to be involved in the mannose-binding pocket, including position 3, 4, 10, 11, 12, 13, 14, 15, 47, 48, 49, 50, 56, 59, 116, 120, 122, 125, 149, 151, 152, 153, 158, 159, 160 and 162. α-Helices were highlighted in dark blue, β-sheets in yellow, β-turns in grey and γ-turns in light blue in the predicted tertiary structure of FimH1.

Based on our findings in the broad host-range serovar Typhimurium, we hypothesized that allelic variation of *fimH* would also associate with host specificity in more host-adapted serovars, potentially contributing to bacterial–host interactions that drive host adaptation. Although most *S. enterica* strains and serovars express type 1 fimbriae and some limited studies suggested *fimH*–host associations^14^,^17^,^18^, no systematic large-scale comparative study has yet linked *fimH* alleles to host-associated strains or serovars. Here, we collected 1,848 individual *fimH* genes from 76 different serovars (Supplementary Data 7), and identified a total of 152 unique *fimH* sequences for 105 different allelic FimH proteins with 166 amino-acid substitution sites and 1 three-nucleotide insertion. The phylogeny of *fimH* demonstrated a strong serovar-specific lineage, with strains of the same serovar clustering either together or in a few distinct branches, as illustrated for the major alleles (Fig. 3). Furthermore, a mutual information analysis confirmed a strong correlation between serovars, *fimH* alleles and FimH proteins, consistent with serovar lineage evolution in *Salmonella*^19^ (Supplementary Fig. 6). Positions of the major substituted amino acids were mapped on a proposed three-dimensional model of the *Salmonella* FimH that is based on the crystal structure of the FimH adhesin on the tip of the *Escherichia coli* type 1 fimbriae^20^,^21^. The model suggests an amino-terminal lectin domain that consists of 174 amino acids and a carboxy-terminal pilin domain of 137–138 amino acids that anchors FimH at the fimbrial tip^20^,^21^,^22^. Among the major allelic FimH proteins, 4 substitutions were detected in the signal peptide, 43 in the predicted lectin domain and 28 in the pilin domain, as predicted from a FimH structural model (Fig. 4a). Interestingly, no mutations were found in the three residues predicted to form the short segment linking the amino-terminal lectin domain of FimH with its carboxy-terminal pilin domain^21^. The predominance of substitutions within the lectin domain in serovar Typhimurium strains (Supplementary Table 4) as well as all other serovar strains studied (Supplementary Table 5), suggested that these mutations were positively selected by recognition of new receptors or affinity maturation of existing receptors. Together, our results support a model of evolutionary adaptation of FimH ligands to host-specific receptors, potentially contributing to host-adaptation and pathogenesis.

### Host-adapted *Salmonella* FimH bind in a host-specific manner

**Figure 5:**
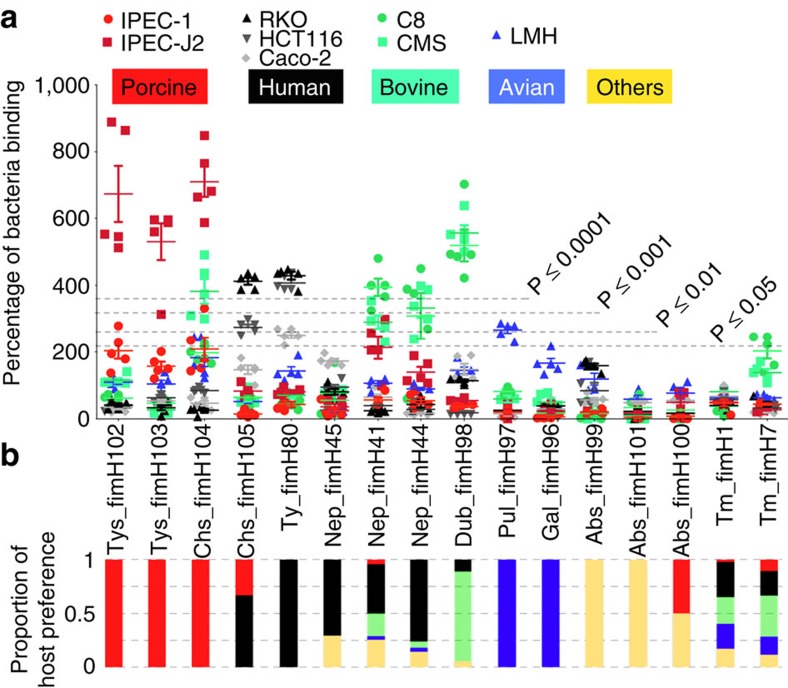
FimH-mediated host-specific bacterial binding to enterocytes or hepato-epithelial cells. (**a**) Recombinant *E. coli* binding mediated by different *Salmonella fimH-*encoded allelic proteins with human (black), porcine (red) and bovine (green) enterocytes, as well as chicken hepatoepithelial cells (blue). The data are expressed as mean percentages of bacterial binding relative to the difference between *fimH2* (100% binding for FimH of *S.* Typhimurium strain AJB3, not shown) and *ΔfimH* (0% binding) with±s.e.m. of five experiments. *P*-values were calculated by the *t*-test that compared groups between each individual alleles and all the rest of data. The null hypothesis assumes a common binding affinity for all variants. The threshold of significance for the *P*-values indicated by grey dash-lines is as follows: 3.58 for *P*≤0.0001; 3.16 for *P*≤0.001; 2.65 for *P*<0.01; 2.19 for *P*≤0.05; and not shown, 1.94 for *P*≤0.1. (**b**) The bars for each *fimH* allele represent the proportion (1 representing 100%) of *Salmonella* isolates from each corresponding host.

To evaluate the biological relevance of the *in silico* determined associations detected above, we assessed the host-specific binding properties of a collection of allelic FimH proteins from major serovars by expressing them in the context of *Salmonella* type 1 fimbriae on recombinant *E. coli*. Binding assays using human, bovine and porcine intestinal epithelial cells, and hepato-epithelial chicken cells, revealed that several allelic FimH proteins conferred significant host-specific binding (Supplementary Figs 7–10). The most impressive host-specific adhesion was exemplified by the *fimH102* or *fimH103* alleles from the porcine-restricted paratyphoid fever *S.* Typhisuis, and *fimH104* of *S.* Choleraesuis, a porcine isolate of this typical porcine-adapted serovar. All three encoded allelic adhesins mediated significantly greater bacterial binding to the porcine enterocytes IPEC-J2 when compared with all the other allelic FimH proteins, including FimH2 of *S.* Typhimurium AJB3, which served as a baseline control (Fig. 5 and Supplementary Fig. 7). Consistent results were obtained with porcine enterocytes IPEC1, albeit the differences were less impressive. In contrast, all three allelic FimH from porcine isolates mediated poor bacterial binding to three human intestinal epithelial cells (Fig. 5 and Supplementary Fig. 8). However, *S.* Choleraesuis can also cause systemic infections in humans, and FimH105 from a human isolate of *S.* Choleraesuis mediated significantly better bacterial binding to the three human enterocytes than the allelic FimH proteins of the three porcine isolates. Conversely, fimbriated bacteria with FimH105 bound poorly to the two porcine intestinal epithelial cells (Supplementary Figs. 7). Notably, the distinct adhesive properties of *S.* Choleraesuis FimH104 and FimH105 were determined by a one amino-acid substitution (V41G), again highlighting the importance of nsSNPs in host specificity.

Both avian-restricted serovars Pullorum and Gallinarium FimH allelic proteins (FimH97 and FimH96, respectively) bound relatively better to chicken cells (Fig. 5 and Supplementary Fig. 9). Both FimH carry the T56I substitution that affects mannose-inhibitable binding^22^, but participates in the avian-specific binding property 17. FimH98 of the bovine-adapted serovar mediated most efficient adhesion to the two bovine intestinal epithelial cells studied. Fimbriated bacteria with the allelic FimH99 and FimH101 proteins of *S.* Abortusovis, and FimH100 of *S.* Abortusequi did not bind to the human, bovine and porcine cells, possibly consistent with a preference for their respective hosts.

Although *S.* Newport has a broad host spectrum, it is frequently isolated from humans affected by foodborne infections, possibly because it is a major serovar isolated from cattle^23^. For this study, most of the *S.* Newport *fimH* sequences investigated were from human isolates, with the *fimH41* and *fimH44* being the most frequent, followed by a few *fimH45* (Fig. 3). Only the former two alleles were present in bovine isolates. This association was consistent with the ability of bacteria expressing the corresponding proteins to bind best to bovine enterocytes (Fig. 5 and Supplementary Fig. 10) as compared with human enterocytes (Fig. 5 and Supplementary Fig. 8). In contrast, the *fimH45* allele was absent from bovine isolates (Fig. 3) and fimbriated bacteria with FimH45 bound best to the human enterocytes, and particularly to Caco-2 cells (Fig. 5 and Supplementary Figs 8 and 10).

Taken together, many allelic variants of FimH demonstrated distinct adherence preferences for host-specific enterocytes or hepatoepithelial cells, confirming the corresponding *in silico* detected associations between allelic adhesins and host specificity in a number of major *Salmonella* serovars. This extended study illustrates for the first time a molecular mechanism that likely contributes to host adaptation. Noticeably, the reported allelic variation of FimH causes a biologically relevant shift in adhesion that occurs both in broad-host range *Salmonella*, such as Typhimurium and Newport, and in host-adapted serovars in support of a significant functional role for nsSNPs in the evolutionary adaptation of the diverse *Salmonella* pathovars.

## Discussion

Most studies of host adaptation by bacterial pathogens such as *Salmonella* focus either on a functional analysis of a specific gene^24^ or utilize genomic comparisons to identify potential virulence genes^4^,^25^ but do not undertake subsequent functional assessments. The systematic approach used here determined that nsSNPs could potentially participate in the strain adaptation of *Salmonella* to individual host species. Our ability to identify specific sequence-determined host-adhesion properties that may contribute to pathodaptation to specific host-species resulted from a novel stepwise approach starting with a genomic comparison of 12 strains of serovar Typhimurium, the quintessential broad host range *S. enterica* serovar. Notably, SNPs in genes for membrane and surface-exposed proteins were among their most differentiating characteristics. In contrast, none of the other strain characteristics, such as the accessory and unique genes, pseudogenes, mobile DNA or genomic rearrangements bore any association with host specificity. A more stringent analysis of genes that encode *S.* Typhimurim surface proteins with known or suspected adhesive properties for colonization of host surfaces clearly highlighted associations between *S.* Typhimurium nsSNPs and specific hosts. This *in silico* result was further supported by the ability of FimH7, the most frequent FimH allelic adhesin of the *Salmonella* type 1 fimbriae from bovine isolates, to preferentially bind to bovine rather than human enterocytes (Fig. 2).

As an expansion of our investigation on FimH variants within another broad-range serovar, FimH of *S.* Newport, further illustrated nsSNPs effects on host-preferences. The bovine isolates of serovar Newport had two *fimH* alleles for proteins that bound best to bovine cells, whereas the third *fimH* was mainly present in human isolates and encoded a FimH adhesin that bound best to human enterocytes (Fig. 5 and Supplementary Figs 8 and 10). In addition, the most impressive host-specific interactions were observed with the FimH adhesins of host-adapted systemic serovars. For example, a detailed analysis of allelic variants of FimH in the swine-adapted serovar Choleraesuis, which can cause systemic diseases in humans, identified allelic adhesins that preferentially bind to intestinal epithelial cells of either humans or swine (Fig. 5 and Supplementary Figs 7 and 10). The host-specific adhesive properties of the FimH allelic proteins corresponded significantly with the host origin of the *Salmonella* strains that carried the respective *fimH* allele, verifying their physiologic relevance *in vivo*. Noticeably, FimH of known host-associated serovars, such as Typhi, Dublin, Gallinarum, Pullorum and Cholereasuis, had at least one *fimH* allele for an adhesin that mediated preferential bacterial binding to the epithelial cells of their respective hosts (Fig. 5 and Supplementary Figs 7–10). Thus, functional analysis of 21 allelic FimH adhesins confirmed the participation of these proteins in host-specific binding, strongly suggesting that preferential adhesion to relevant mammalian or avian cells participates in the evolutionary adaptation to specific hosts, even within individual serovars. These relevant associations were detected despite potential confounding effects, such as the likely inclusion of *Salmonella* strains from broad host serovars transiently passaging in non-preferential hosts, the polygenic nature of host-adaptive evolution, or nsSNPs responsible for neutral substitutions, highlighting the power of the approach.

Our finding that all mammal-specific FimH allelic proteins identified bound intestinal cells of their hosts in a mannose-inhibitable manner was somewhat surprising, as it is currently unclear how binding of mannose residues on glycoprotein receptors could contribute to host specificity. We predict that the differential ability of FimH adherence to bind to various host cells is due to the density, accessibility, flexibility, orientation or length of the diverse mannose-bearing oligosaccharides on host cell-specific receptors^18^,^26^. Indeed, such factors may explain why simple *in vitro* binding assays that classify FimH adhesins according to their binding affinity for mono-, tri- or penta-mannose model receptors, or adhesion to yeast or non-intestinal human cell lines^27^,^28^,^29^,^30^,^31^ do not always reflect their host-specific binding to intestinal cells. For example, we found that the serovar Typhisuis FimH, which is identical to the serovar Indiana FimH reported to have low-affinity binding *in vitro*^29^, bound strongly to the porcine enterocytes (Fig. 5 and Supplementary Fig. 7). Similarly, Choleraesuis FimH105 (V41G) and Newport FimH45, which do not bind mannose *in vitro*^29^, bound well to human intestinal cells, with the latter binding best to human Caco2 cells (Fig. 5 and Supplementary Fig. 8). Thus, our results support the critical importance of utilizing physiologically relevant cells to functionally characterize pathogen–host interactions.

**Figure 6:**
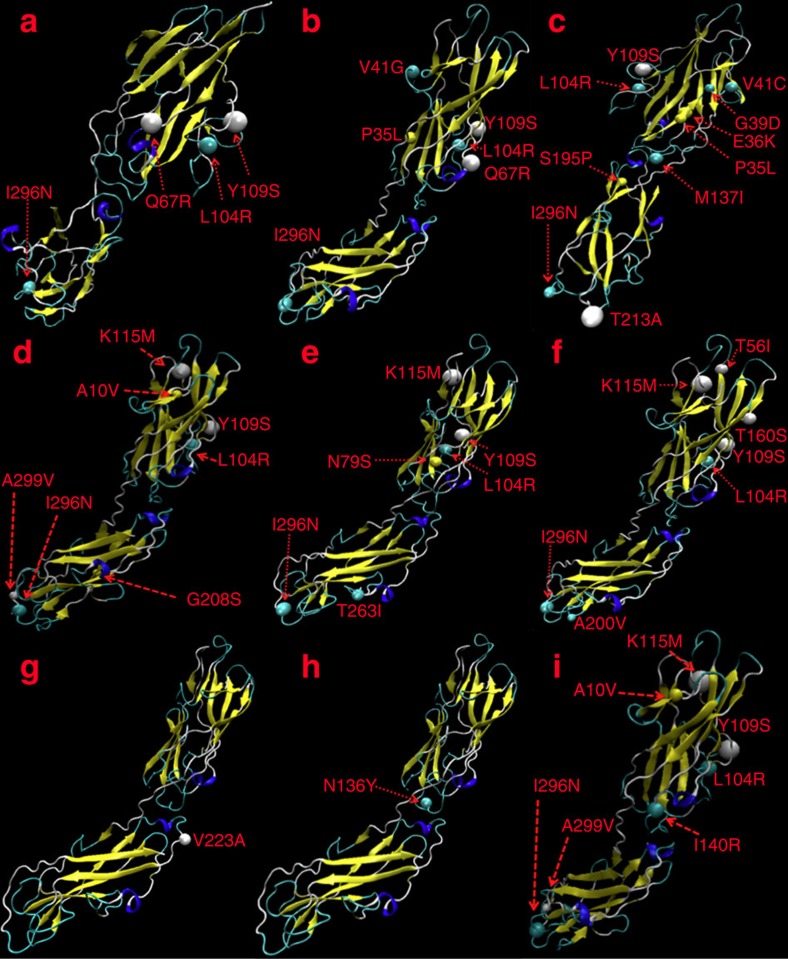
Substituted sites on FimH alleles visualized on the *Salmonella* FimH model (*S.* Typhimurium SL1344). The balls highlight the substituted residues. (**a**) Serovar Typhisuis FimH102 or FimH103; (**b**) serovar Choleraesuis FimH105; (**c**) serovar Typhi FimH80; (**d**) serovar Newport FimH44; (**e**) serovar Dublin FimH98; (**f**) serovar Gallinarum FimH95; (**g**) serovar Typhimurium FimH7; (**h**) serovar Typhimurium FimH2; (**i**) serovar Abortusequi FimH100. α-Helices were highlighted in dark blue, the β-sheets in yellow, the β-turns in grey and the γ-turns in light blue in the predicted tertiary structure of FimH1.

Although the binding of all mammalian FimH allelic proteins is mannose-sensitive, the specific binding of FimH adhesins from avian Pullorum and Gallinarum to chicken leukocytes is not mannose-inhibitable, indicating that this FimH allele recognizes a distinct receptor^17^. Here, we confirmed the avian-specificity and relative insensitivity to mannose inhibition of the Gallinarum and Pullorum FimH, with the incomplete inhibition observed likely reflecting the use of alpha-methyl-D-mannoside, a modified and powerful inhibitor, in excess (Fig. 5 and Supplementary Fig. 9). Notably, the FimH amino-acid substitution T56I in Gallinarum, Pullorum and chicken isolates of Paratyphi B with the *fimH56* allele (Figs 3, 4b and 6f) is a strong determinant for avian specificity^17^,^22^,^29^,^32^. Mutation of the avian *fimH* to substitute an isoleucine with a threonine at position 56, restores its ability to bind a mannosylated glycoprotein^22^, and a Gallinarum strain engineered to express such a mannose-binding FimH was significantly less invasive in chicks^32^, supporting the idea that this single amino-acid substitution determines both mannose-binding and avian host specificity. In addition, Gallinarum and Pullorum adhered to and invaded mammalian cells only when engineered to express the Typhimurium type 1 fimbriae with a threonine in position 56 of FimH^33^. Taken together, our results corroborate the importance of the FimH amino-acid substitution at position 56 to make a jump between mammalian and avian hosts. Further, our data confirm that a single amino-acid substitution in the binding pocket of FimH permits a switch in host specificity based on the presentation of mannose or non-mannose receptors by mammalian or avian hosts, respectively.

Although nsSNPs were found throughout mammalian-adapted FimH sequences, they were more abundant in the lectin domain, many surrounding the binding pocket (Fig. 4c), suggesting a direct role in binding. A comparison of these sequences identifies amino acids that most likely contribute to host-specific binding. For example, substitutions in the lectin domain, such as E36K and V41C of the Typhi FimH80 likely alter the conformation of the binding pocket to promote human specificity (Figs 3 and 6c). FimH80 also shares a substituted V41 with Choleraesuis FimH105 (albeit V41C versus V41G) from a human isolate, suggesting that position 41 of FimH has a role in human specificity (Fig 6a–c). Similarly, the Q67R substitution likely promotes host adaptation of porcine-associated serovars such as Typhisuis and Choleraesuis, but not of bovine-associated serovars such as Dublin (Fig. 6e) and Newport (Fig. 6d), with the exception of the human-adapted FimH45 of Newport (Fig. 3). Thus, a selective group of amino-acid residues within the FimH lectin domain appears to play a dominant role in determining host-specific binding by the adhesin. Moreover, residues in these positions must influence FimH binding indirectly, as they are not found within the binding pocket.

Most surprising were variable FimH amino-acid residues that resided outside of the lectin domain but still affected host-specific adhesion. For example, several variable residues around the linker domain increased binding to mannose, as confirmed with the natural FimH2 N136Y substitution (Figs 3 and 6h)^17^,^34^. Linker domain substitutions in natural FimH also modulated host specificities, as shown with the V223A substitution of FimH7, which increased the binding affinity for bovine cells (Fig. 6g). Similarly, the I140L substitution in FimH100 of Abortusequi abrogated the binding to bovine cells detected with Newport (Fig. 6d,i). To a certain extent, these findings are consistent with a previous random mutagenesis study that revealed the role of the linker domain in allosteric effects on FimH-mediated adhesion^34^,^35^. When FimH is subjected to tensile force, it undergoes structural changes in which an extended linker domain allows the pilin and lectin domains to separate, forming a binding pocket that closes around the mannose receptor like a Chinese finger trap^20^. Thus, one can speculate that residue substitutions in the linker domain could indirectly lead to conformational changes in the binding pocket to influence FimH host-specific binding. Finally, it is possible that stepwise mutations, some near the linker domain, participate in the evolution of host adaptation with the addition of individually silent mutations affecting adhesion in a cumulative or epistatic manner.

Collectively, our results on the function of FimH variants illustrate the significance of nsSNPs as a molecular mechanism by which *Salmonella* expands its host range. Most significantly, this study provides the first use of a genome-wide association study to prompt a subsequent systematic functional analysis, which revealed an evolutionary positive selection process that may contribute to host adaptation of *Salmonella*. Although our results need *in vivo* confirmation using corresponding animal models, our current *in vitro* identification of host-specific allelic variants in surface molecules should provide the basis for future diagnostic assays of host-specific pathogens and may allow for the development of anti-adhesive antimicrobials that interfere with host-specific intestinal colonization and invasion. Moreover, this study opens the possibility that allelic variation in a wide range of bacterial proteins that participate directly in virulence, or indirectly by modulating metabolic or regulatory pathways^10^ may also contribute to host specificity and pathogenesis. As such, this work has broad implication in the field of bacterial pathogenesis, as the used approach can identify and assess the role of specific allelic variants in any pathogen for which groups of isolates with relevant metadata and appropriate functional tests exist.

## Methods

### Bacterial strain and data collection

A total of 382 *Salmonella* Typhimurium strains were isolated in the United States America between 1988 and 2010 from different hosts or from the environment. Human and food isolates were obtained from collections at the US Centers of Disease Control and Prevention (CDC) and the US Food and Drug Administration (FDA), respectively, whereas animal isolates were from the *Salmonella Reference Center* at the University of Pennsylvania (UPENN). All the *Salmonella* Typhimurium isolates were identified by standard serotyping methods, using O- and H-antigen agglutination, based on the Kauffmann–White Scheme^36^. Isolates were grown on LB (Lennox) medium, and single colonies were incubated in LB broth overnight at 37 °C. Bacterial cells were pelleted by centrifugation (3,700*g* for 10 min) and DNA was extracted using the Wizard SV 96 Genomic DNA Kit (Promega) according to the manufacturer’s instructions. DNA quality and quantity were evaluated by gel electrophoresis and determined with a NanoDrop 1,000 spectrophotometer (Thermo Fisher Scientific). Genomic templates were normalized to 5 ng μl^−1^ for targeted massive parallel sequencing, as described below. An additional 12 complete genomes with metadata of serotype Typhimurium were downloaded from GenBank and sequence data from 186 Typhimurium genomes were collected from the NCBI SRA database. All 580 Typhimurium isolates are listed in Supplementary Tables 3 and 4. A total of 1,268 additional individual *fimH* gene sequences from 76 serovars with metadata of the corresponding strains were extracted from six publications^29^,^37^,^38^,^39^,^40^,^41^ as well as new sequences produced from our lab.

### DNA sequencing

For targeted massive parallel sequencing, primer pairs for 15 genes, including the genes for 12 fimbrial adhesins (StcD, SafD, BcfD, FimH, StbD, SthE, StdD, StiH, StfH, LpfD, StjA, PefA)^21^ and 3 outer-membrane proteins (OmpA, OmpC, OmpN) were designed and synthesized (Integrated DNA Technologies, Inc.) with 3–4 primer pairs per gene (Supplementary Table 6). The sequencing libraries were prepared using the Access Array system (Fluidigm South San Francisco)^14^. Quality and quantity of the amplicon libraries were evaluated with a 2,100 Bioanalyzer instrument (Agilent Technologies) and NanoDrop. The libraries were pooled in equal amounts for pyrosequencing with a 454 GS FLX sequencer using Titanium chemistry (454 Life Sciences, Roche) at the DNA Sequencing Facility of UPENN. An in-house Perl script was used for sequence splitting and barcode removal. Sequence assembly and mapping were done with SeqMan (DNASTAR, Inc.). A total of 15 genes of 382 strains were sequenced with a coverage of more than 30 and a Phred quality score of more than 40 for data analysis. For Sanger DNA sequencing, the *fimH* gene of 210 clinical isolates from various *S. enterica* serovars were amplified with the Pfu polymerase (New England Biolabs Inc.) and each individual gene sequence was assembled using at least three sequencing reads to get a Phred quality score of more than 30.

### Genomic analysis

Mauve^42^ was used for comparative analysis of 12 sequenced Typhimurium full genomes. A core genome was assigned by using thresholds of 95% sequence identity and 95% sequence length coverage. Comparative circle map and gene functional categories were determined by the rapid annotation of microbial genomes using subsystems technology^43^. *P*-value associated with a functional category measured the likelihood that the association between a set of genes with SNPs and a given functional category is due to random chance. The smaller the *P*-value, the less likely the association was random and the more significant the association. In general, *P*-values less than 0.05 indicated a statistically significant, non-random association. *P*-values were calculated using the right-tailed Fisher exact test (Prism, GraphPad Software, Inc.). All proteins with less than 50 amino-acid residues were removed from the core genome and pangenome analysis. The phage genes from the Typhimurium genome were determined by PHAST^44^. All the pseudogenes were assigned by original genome annotation of the 12 complete Typhimurium genomes under manual correction. A total of 186 strains of Typhimurium with SRA data produced by Illumina paired-end technology were submitted for *de novo* genome assembly and annotation by using CloVR^45^. CloVR used both virtual machine and cloud computation technology for high-throughput data processing. All the annotated genomic data were uploaded into SEED^43^ for further data storage, sequence extraction and analysis. The 15 genes studied for all the 186 strains had quality score of more than 30. SNPfinder^46^ was used to detect the SNPs for the 12 *S.* Typhimurium complete genomes.

### Population and phylogenetic analysis

DnaSP5 (ref. 47) was used to estimate several measures of DNA sequence variation within and between populations, including neutrality analysis (Tajima’s D) and SNP detection. Nucleotide diversity estimates^48^ and recombination analyses were carried out by using DataMonkey^49^. The homoplasious SNPs, that is, SNPs due to recombination and horizontal gene transfer, were detected by using START2 (ref. 50) with a threshold of 0.6.

### Statistics and association analysis

All the SNPs and their corresponding metadata were used for association studies. The Akaike Information Criterion (AIC) of the multinomial logit (logistic regression) model was used for testing the genetic association of multiple phenotypes with the ‘nnet’ R package^15^ and the following equation: AIC*=−*2 Log *L+*2((*k*−1)*+s*), where *k* is the number of levels of the dependent variable and *s* is the number of predictors in the model. The model with the smallest AIC was considered the best. The resulting association coefficient *e* was determined as followed: **e***=(y ft)(yt f)*^*⁁*^*-1* (*y*=number of correct predictions, *yt*=total number of correction prediction, *f*=number of false prediction, *ft*=total number of false prediction). MultiPhen^16^ identified the linear combination of traits most associated with each genetic variant by applying a reversed ordinal regression, such that genotype (allele count) is regressed on a collection of traits. The test for association was a likelihood ratio test for model fit, testing whether all regression coefficients in the model were jointly significantly different from zero. RF was used to identify key SNPs involved in the studied associations (SPM v7.0, Salford Systems). The three-dimensional scatterplot (Fig. 1b and Supplementary Fig. 4) of the multi-dimensional scaling coordinates were obtained from the RFs proximity matrix. All heat-map images were produced by using the neighbour-joining method for hierarchical clustering of MeV with 1,000 bootstrap replicates^21^. Multivariate mutual information statistics was used to detect and evaluate the dependency among *fimH* alleles, FimH alleles, serovars and host origins^51^. The maximum likelihood estimators of Shannon’s entropy and multiple mutual information were obtained by sampling the system variables and using the resulting empirical values of probability distributions.

### Structure-function analysis

Processed FimH allele 1 (FimH1) from strain SL1344, which comprises 291 amino acids (or 313 residues minus its 22 residue long signal peptide), was used for modelling the structure. The secondary structure of FimH was predicted by machine learning with the I-TASSER server^52^. The best template structure was identified by matching the protein sequence and predicted secondary structure with the solved structure (1klf, PDB library), using LOMET threading^53^. The mannose-binding site was mapped by matching the predicted structure with structures of the PDB function library using COACH^54^. The structure was edited and visualized with VMD^55^. The functional data for binding properties to mannose receptors were from the following papers^17^,^18^,^29^.

### Site-directed mutagenesis and recombinant engineering

The construction of recombinant *E. coli* that express different *Salmonella fimH* alleles was done as follows. Different *fimH* alleles were amplified using the high-fidelity Pfu DNA polymerase with appropriate primers (Supplementary Table 14) and templates from the desired strains, restricted with *Nde*I and *Bam*HI, and ligated to the correspondingly restricted pMAL-c2X plasmid (New England Biolabs, Inc.). All the *fimH* were sequenced by Sanger Sequencing to confirm correct amplification (UPENN, DNA Sequencing Facility). *E. coli* AAEC189, which lacks the *E. coli fim* gene cluster^56^and carries plasmid pAZ37 that contains the *S.* Typhimurium *fim* gene cluster with no functional *fimH*^17^, was used to transform with plasmids expressing the different *Salmonella* FimH alleles. Expression of the type 1 fimbriae and mannose-specific recognition by FimH were determined by slide agglutination with anti-type 1 fimbriae antisera (seroagglutination) and by yeast cell (*Saccharomyces cerevisiae*) aggregation, respectively. The site-directed mutant of *fimH*, *fimH80* was used as template for overlap PCR with primer pairs (forward: 5′-AAATGTACCAACGCCGCGGCGCAGGCCTATTTATC-3′; reverse: 5′-GATAAATAGGCCTGCGCCGCGGCGTTGGTACATTT-3′) targeting the corresponding position. The mutated *fimH* amplicon was cloned into pMAL-c2X, sequenced to confirm correct amplification and introduced into AAEC189.

### Eukaryotic cell cultures

The human colonic cell lines Caco-2 (ATCC HTB-37), HCT116 (ATCC CCL247) and RKO (ATCC CRL2577) were obtained from the NIH/NIDDK Digestive Diseases Center at UPENN. All three cell lines were routinely cultured in Dulbecco’s Modified Eagle Medium (DMEM; Invitrogen, Life Technologies) supplemented with 20% (v/v) fetal bovine serum (FBS), 1% (v/v) non-essential amino acids and antibiotics to a final concentration of 100 U ml^−1^ penicillin and 100 μg ml^−1^ streptomycin (Gibco, Life Technologies). The porcine cell lines IPEC-1 (DSMZ ACC 705) and IPEC-J2 (DSMZ ACC 701)^57^,^58^ were cultured with 10% FBS (Sigma-Aldrich), 1% penicillin/streptomycin, 1% insulin/transferrin/selenium (Gibco), and 5 ng ml^−1^ epidermal growth factor (Sigma) in DMEM/F-12/HAM (1/1/1, v/v/v; Gibco). The bovine intestinal epithelial cells were immortalized with the thermo-sensitive mutant of a retrovirus vector coding for the SV40 large T-antigen oncogene, using standard procedures, after isolation by enzymatic (C8, J8 and J8A2 cell lines) or Matrisperse dissociation (CMS cell line) of the intestinal mucosal segments^59^,^60^,^61^ (personal communication from Jacques Mainil). The cells were seeded on the surfaces of plates pretreated with bovine collagen (95% type I, Vitrogen 0701/FXP-019, 1 μg cm^−2^; Nutacon BV), using OptiMEM medium (Gibco) with 1% (v/v) of a mixture of antibiotics and antimycotic (Gibco), 0.2% of bovine pituitary gland extract (Gibco), 1% of insulin-transferrin-selenium (Gibco), 1% of glutamax (Gibco), 1% of sodium pyruvate (Sigma), 10 nM hydrocortisone (Sigma), 20 nM triiodothyronine (Sigma), 10 ng ml^−1^ epidermal growth factor (Sigma), 10 μg ml^−1^ linoleic acid-albumin from bovine serum albumin (Sigma), 1% (v/v) nonessential amino acids (Gibco) and 1% (v/v) FBS (Hyclone). The avian cell line LMH (ATCC CRL-2117™) was seeded on 0.1% gelatin-coated plate and cultured in Waymouth’s MB 752/1 medium (Gibco) with 10% (v/v) FBS and antibiotics (100 U ml^−1^ penicillin and 100 μg ml^−1^ streptomycin, Gibco). All the cells were incubated at 37 °C in a humid atmosphere containing 5% of CO_2_ or 10% CO_2_ for the bovine cells.

### Bacterial binding assays

Human, porcine, bovine and chicken epithelial cell cultures were used for the binding assays with recombinant *E. coli* AAEC189 (Δfim_E.coli_) expressing *Salmonella* type 1 fimbriae with different FimH alleles. The bacteria were grown under static condition for 48 h. All the bacteria were washed three times with PBS, and diluted in DMEM to 10^7^ CFU ml^−1^ before use. The overnight seeded eukaryotic cells were grown to monolayers in 96-well plates (Corning, CLS3596). Bacteria were added at a multiplicity of infection of 200 to 1 and allowed to interact with the cells for 1 h at 37 °C in 5% CO_2_. The cells were then washed five times with PBS and lysed with 1% Triton (Sigma). The number of CFU in each well was quantified by plating serial dilutions of cell lysates on LB plates. For inhibition, bacterial binding was tested in the presence of 50 mM methyl-D-mannopyranoside (α-mm). Binding data were the results of five individual repeated experiments. Bacterial binding was compared with the binding of recombinant *E. coli* that expressed the FimH3 (FimH of strain AJB3) set at 100% and the binding of recombinant bacteria that expressed no *fimH*, set at 0%. Percentage of bacterial binding mediated by FimH allele X (X representing any specific FimH allele studied) was calculated in the following way: % bacterial binding with **X***=*(CFU_X_−CFU_AJB3_)(CFU_AJB3_−CFU_ΔfimH_)^⁁^-1. The *P*-value was calculated by the *t*-test that compared groups between each individual alleles and all the rest of data. The null hypothesis assumed a common binding affinity for all variants.

### Analysis of fimbriae expression

Recombinant fimbrial expression on *E. coli* was determined by two methods. First, a semi-quantitative standard slide seroagglutation test with anti-fimbrial antisera was used to check for fimbriation^17^. Second, the relative amount of fimbriae on bacteria was measured quantitatively by two parallel twofold serial dilution ELISAs, one assay measuring the level of bacterial fimbriation, and the other controlling the level of coated bacteria in the plastic wells. For this, fimbriated *E. coli* AAEC189 expressing *Salmonella* type 1 fimbriae were washed three times in PBS, adjusted to an OD600 of 1.0 and coated on 96-well microlitre plate, followed by blocking with 1.5% BSA. Bacterial coating efficiency was quantitated with biotinylated anti-*E.* coli antibodies (10^−2^, Pierce PA1-73035, Thermo Fisher Scientific) and the amount of expressed fimbriae, with anti-fimbriae antisera (5 × 10^−2^) followed by Streptavidin HRP conjugate (Pierce) or ECL anti-rabbit IgG HRP (2 × 10^−3^, GE Healthcare Bio-Sciences, RPN4301), and 1-Step Turbo TMB-ELISA Substrate Solution (Pierce), with standard PBS washes in-between each step. Reactions were stopped with 2 M H_2_SO_4_ and absorbance (*A*=450 nm) was determined with a Synergy HT Multi-Mode Microplate Reader (BioTek). Anti-bacteria and -fimbriae antibody dilutions giving 50% binding (*A*_50_) for each fimbriated bacteria were used to calculate relative amounts of fimbriae per bacteria (fimbria_x_ (*A*_50_))(bacteria_x_ (*A*_50_))^⁁^-1. The experiments were repeated three times.

## Additional information

**Accession codes:** All the sequence data produced in this study were submitted to GenBank under accession number PRJNA297164.

**How to cite this article:** Yue, M. *et al.* Allelic variation contributes to bacterial host specificity. *Nat. Commun.* 6:8754 doi: 10.1038/ncomms9754 (2015).

## Supplementary Material

Supplementary Information Supplementary Figures 1-11, Supplementary Tables 1-6 and Supplementary References.

Supplementary Data 1 List of orthologous proteins encoded by Salmonella Typhimurium plasmids.

Supplementary Data 2 List of Salmonella Typhimurium strains from GenBank and SRA database (NA, information is not available)

Supplementary Data 3 List of Salmonella Typhimurium isolates from the University of Pennsylvania Salmonella Reference Center (Code A), Pennsylvania Department of Health (Code B), U.S. CDC (Code C) and U.S. FDA (Code D).

Supplementary Data 4 List of SNPs with host associations detected by random forest.

Supplementary Data 5 List of SNPs with host associations detected by AIC of multinomial regression.

Supplementary Data 6 List of SNPs with host associations detected by mPhen (cells highlighted in yellow indicate association that have a correlation coefficient with P-value < 0.01).

Supplementary Data 7 List of isolates with fimH sequences from Salmonella enterica subsp. I ("databank" refers to the Salmonella genome database at http://www.ncbi.nlm.nih.gov/genome/152).
