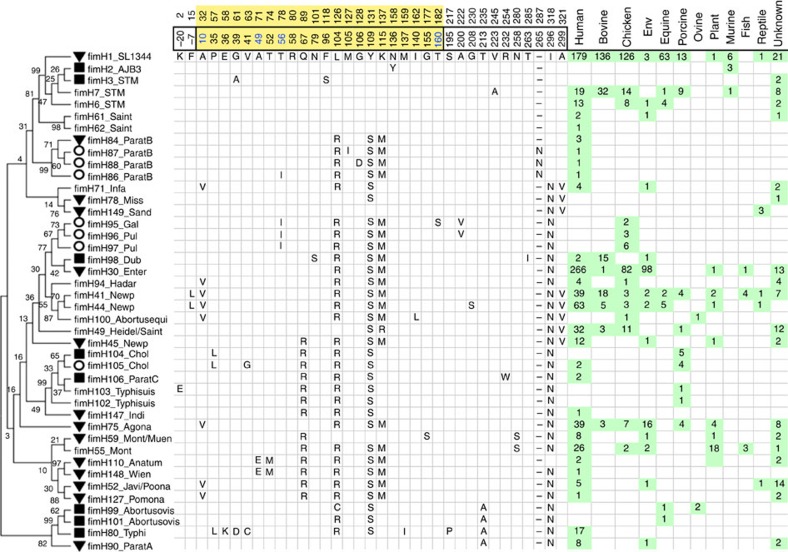# Corrigendum: Allelic variation contributes to bacterial host specificity

**DOI:** 10.1038/ncomms15229

**Published:** 2017-08-08

**Authors:** Min Yue, Xiangan Han, Leon De Masi, Chunhong Zhu, Xun Ma, Junjie Zhang, Renwei Wu, Robert Schmieder, Radhey S. Kaushik, George P. Fraser, Shaohua Zhao, Patrick F. McDermott, François-Xavier Weill, Jacques G. Mainil, Cesar Arze, W. Florian Fricke, Robert A. Edwards, Dustin Brisson, Nancy R. Zhang, Shelley C. Rankin, Dieter M. Schifferli

Nature Communications 6 Article: 8754 ; DOI: 10.1038/ncomms9754 (2015); Published: 10 30 2015; Updated: 08 08 2017

In Fig. 3 of this Article, the numbers of isolates studied for the *fimH41*_Newp, *fimH44*_Newp and *fimH45*_Newp alleles were inadvertently swapped. The correct version of Fig. 3 appears below as Fig. 1.

## Figures and Tables

**Figure 1:**