# Supplementary material for: Allelic variation contributes to bacterial host specificity
Source: Nat Commun. 2015 Oct 30;6:8754. doi: 10.1038/ncomms9754 (PMC4640099; doi:10.1038/ncomms9754)
Supplement: Supplementary Information — Supplementary Figures 1-11, Supplementary Tables 1-6 and Supplementary References. [file ncomms9754-s1.pdf]

Supplemental Figures

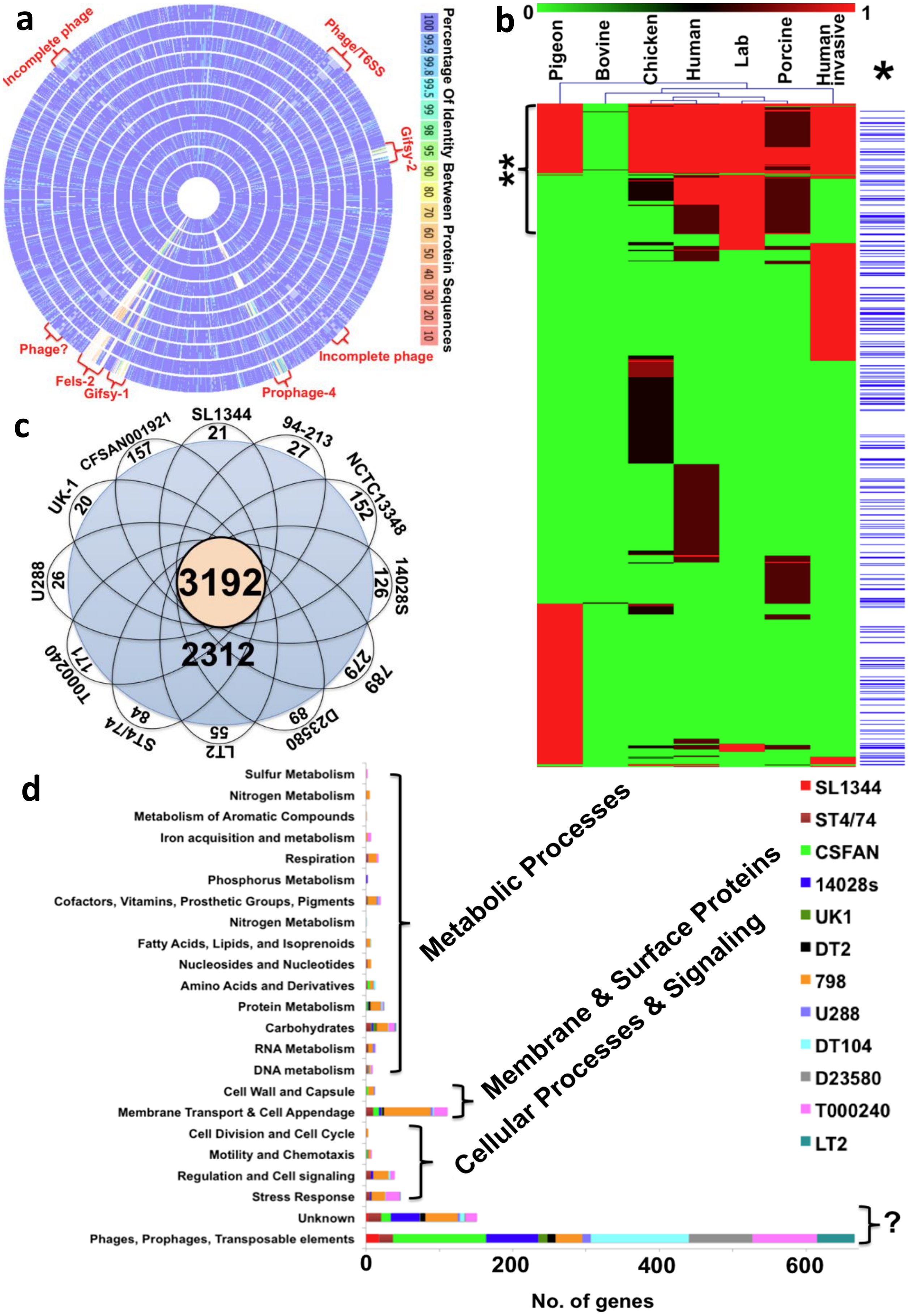

Supplemental Figures

Supplementary Fig. 1. Comparative analysis of 12 *S*. Typhimurium genomes. (a) Comparative circle map of 12 complete *S*. Typhimurium genomes; the SL1344 genome served as the reference for 11 Typhimurium genomes; from the inner to the outer circle, 94-213, NCTC13348, 14028s, 798, D23580, LT2, ST4/74, T000240, U288, UK-1, and CFSAN001921; the color bar on the right indicates the percentage of protein sequence identity against the reference genome; high variability regions corresponded to the eight phage regions of the reference genome, as labeled in red. (b) Heat map representing a clustering profile of 901 genes with nonsynonymous substitutions (NS) for the core genome of the 12 *S*. Typhimurium (with SL1344 as the reference strain), grouped by origin of isolation (7 groups). The green and red color bars represent numbers of detected NS per isolate from each host origin groups. (*) Each blue bar on the right side highlights a gene belonging to the membrane transport and cell appendages, and the cell wall and capsule categories. (**) The bracket on the left side shows clustering groups with hot spot substitutions and significant numbers of genes for surface-exposed proteins. (c) Flower plot illustrating the numbers of fully shared genes (3192 core genes), partially shared genes (2312 accessory genes), and unique genes (varying from 20 to 279 genes) for the 12 strains. (d) A histogram profile of functional categories for 1207 unique genes in 12 *S*. Typhimurium. Each color represents a different isolate (SL1344, DT2=94-213, DT104=NCTC 13348, 14028s, 798, D23580, LT2, ST4/74, T000240, U288, UK1=UK-1, and CFSAN=CFSAN001921). Four major groups of genes are highlighted, namely for metabolic processes (170 genes from 15 functional categories, 14.06% of total unique genes), membrane and surface proteins (123 genes from 2 functional categories, 10.19% of total unique genes), cellular processes and signaling (97 genes from 4 functional categories, 8.04% of total unique genes) and genes encoding proteins with unknown functions (817 genes from 2 functional categories, 67.69% of total unique genes). The latter group of genes was the largest, with many of its genes related to phage acquisition.

Supplemental Figures

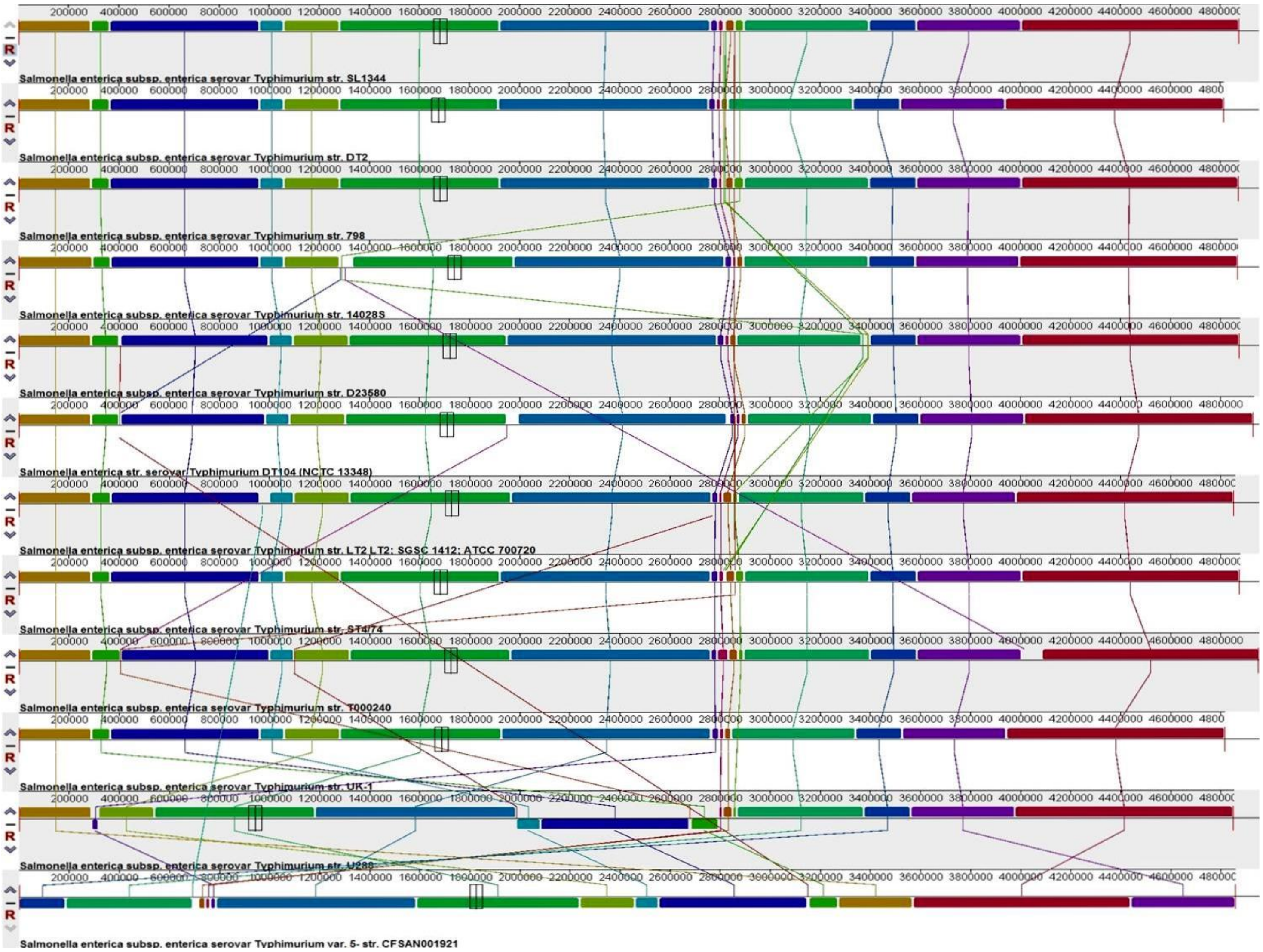

Supplementary Fig. 2. Comparison of the genomic structures of the 12 *S*. Typhimurium maps. The maps shown from top to bottom are for SL1344, DT2 (94-213), NCTC 13348, 14028s, 798, D23580, LT2, ST4/74, T000240, U288, UK-1, and CFSAN001921. Regions with same color represent homologous fragments identified by Mauve. Beside genomic recombinations and rearrangements, comparison of the 12 *S*. Typhimurium maps shows little genomic structural variation for this serovar.

Supplemental Figures

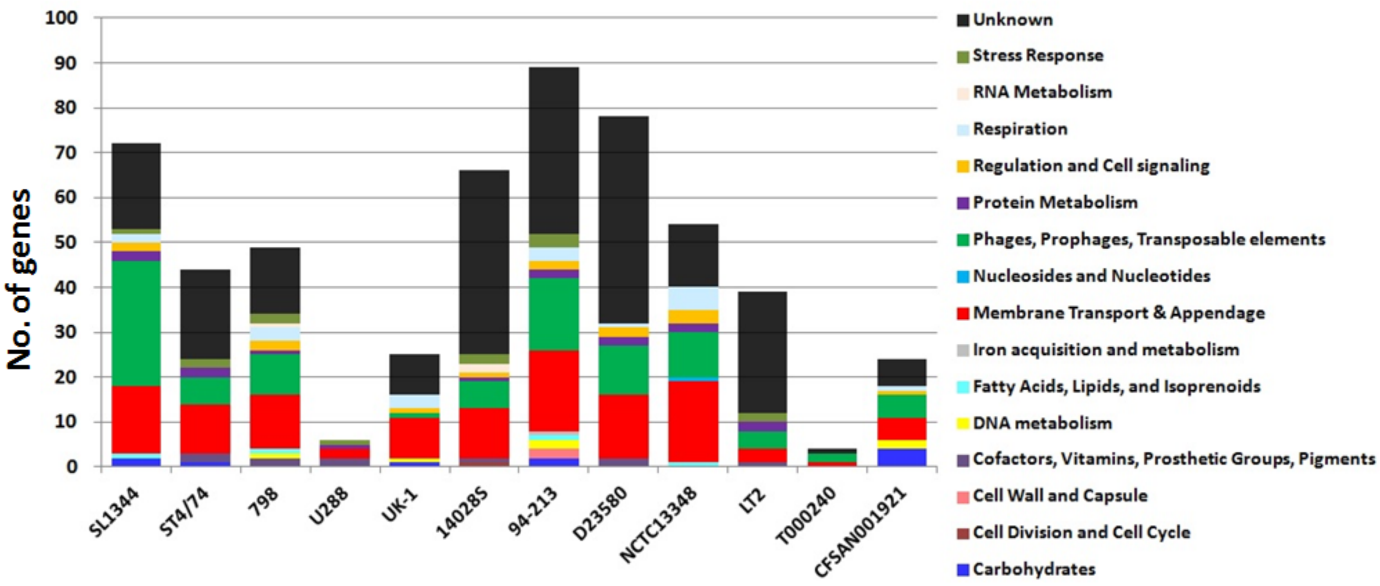

Supplementary Fig. 3. Distribution of pseudogenes divided in functional categories among 12 *S*. Typhimurium strain. Host-adapted serovars (e.g. the pigeon-adapted isolate 94-213 and the human-adapted isolate D23580) had the most pseudogenes. Most of these pseudogenes had an unknown function, a phage-related origin, or were in the category of membrane transport and cell appendage pseudogenes. Column segments with the same color represented different functional categories, as determined by the RAST database.

Supplemental Figures

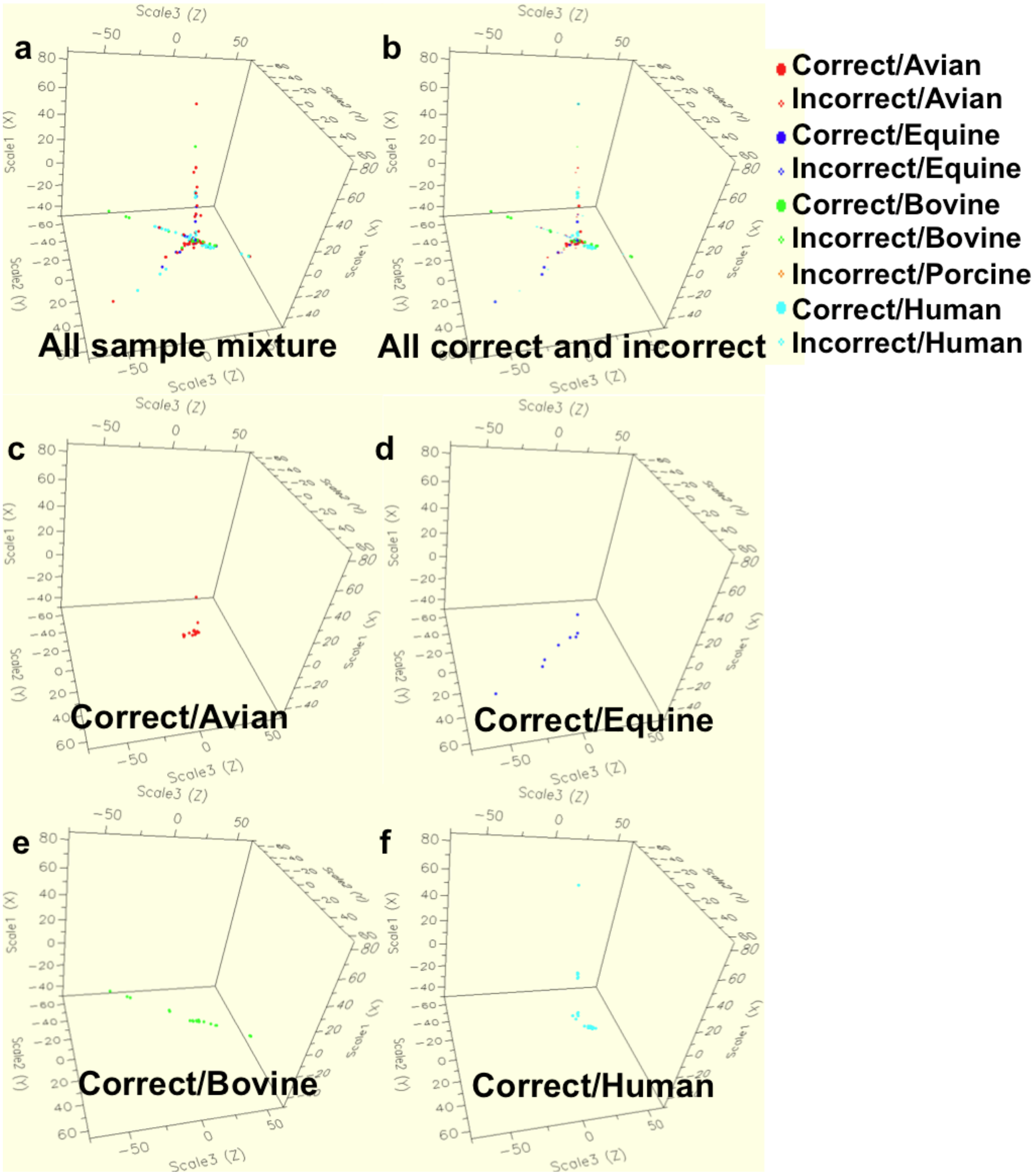

Supplementary Fig. 4. Six 3-dimensional scatterplots of the scaling coordinates obtained from the random forests proximity matrix. The colored dots in the scatterplots correspond to strains of different host origins (red for avian, dark blue for equine, dark green for bovine, pink for porcine, light blue for human). (a) All strains from the five host origins. (b) Correct (large solid dot) and incorrect predictions (small hollow dot) of the random forest analysis of all the strains. Correct predictions for the (c) avian, (d) equine, (e) bovine, and (f) human isolates.

Supplemental Figures

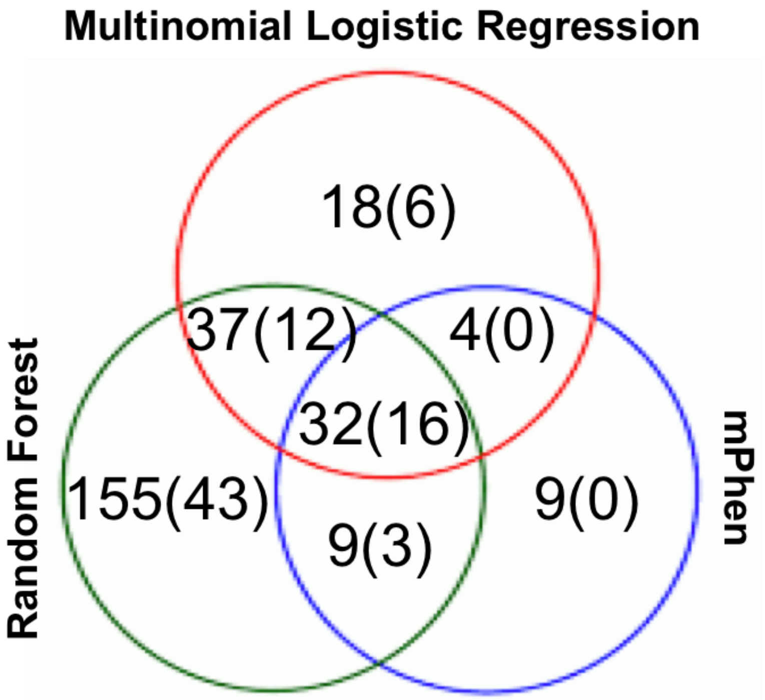

Supplementary Fig. 5. Venn diagram of the detected SNPs (nsSNPs in parenthesis). Significant host-specific associations were detected by using three different approaches (AIC of multinomial regression, random forest and MultiPhen).

Supplemental Figures

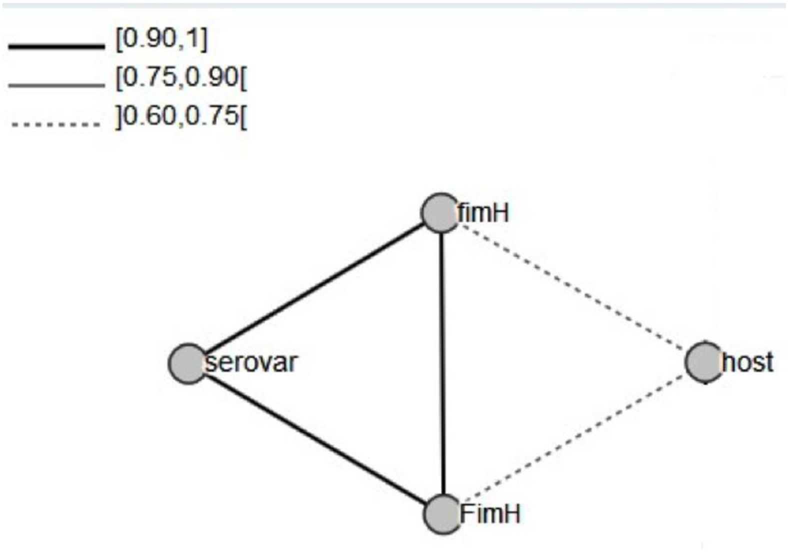

Supplementary Fig. 6. Association levels between serovars, *fimH* SNPs, FimH alleles and host origins. Multivariate mutual information statistics was used to detect and evaluate the dependencies. As expected, results show a strong correlation between serovars *fimH* and FimH alleles. A weaker but significant correlation was detected between host origins and both *fimH* and FimH alleles. The Shannon entropies or mutual information indexes that are shown by three different types of bars for skewed distributions are estimates of parameters of dependencies among the different variables.

Supplemental Figures

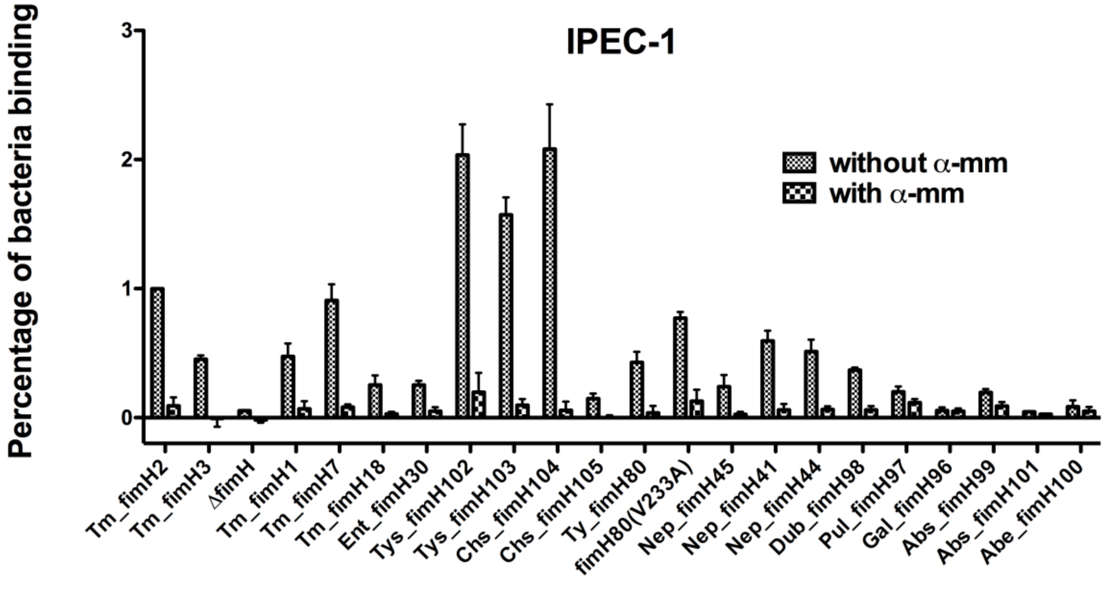

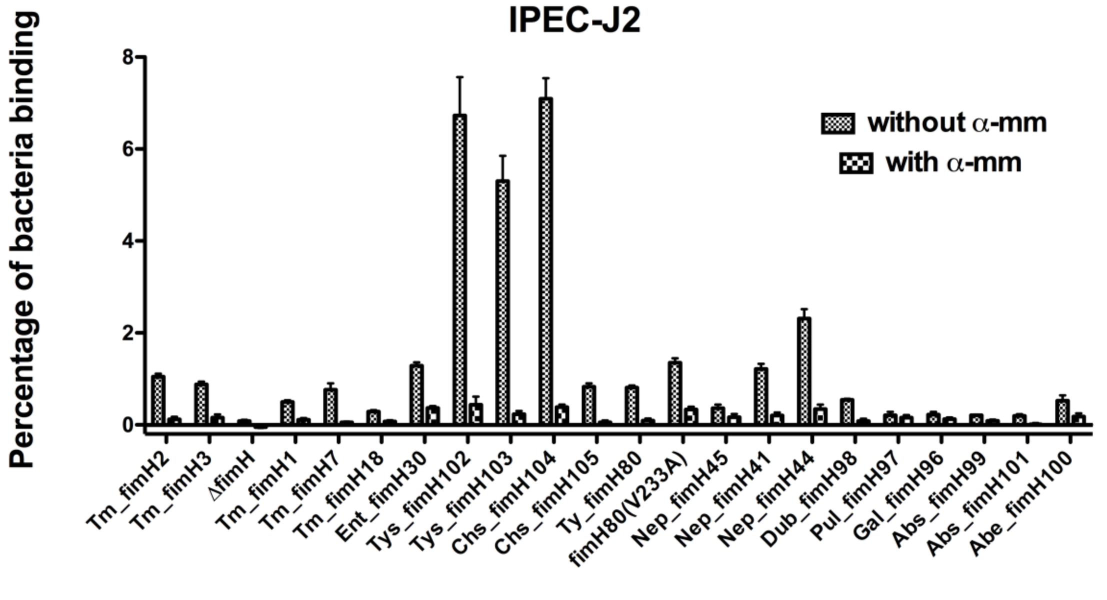

Supplementary Fig. 7. Bacterial adhesion to porcine intestinal epithelial cells. Binding of recombinant *E. coli* expressing *Salmonella* type 1 fimbriae with different FimH alleles, as listed in Supplementary Fig. 9. Two porcine intestinal epithelial cell lines (IPEC-1 and IPEC-J2) were used for the binding assays. The data are averages with SEM from at least four replicated assays. All the binding assays were done in the presence or absence of methyl-D-mannopyranoside (α-mm).

Supplemental Figures

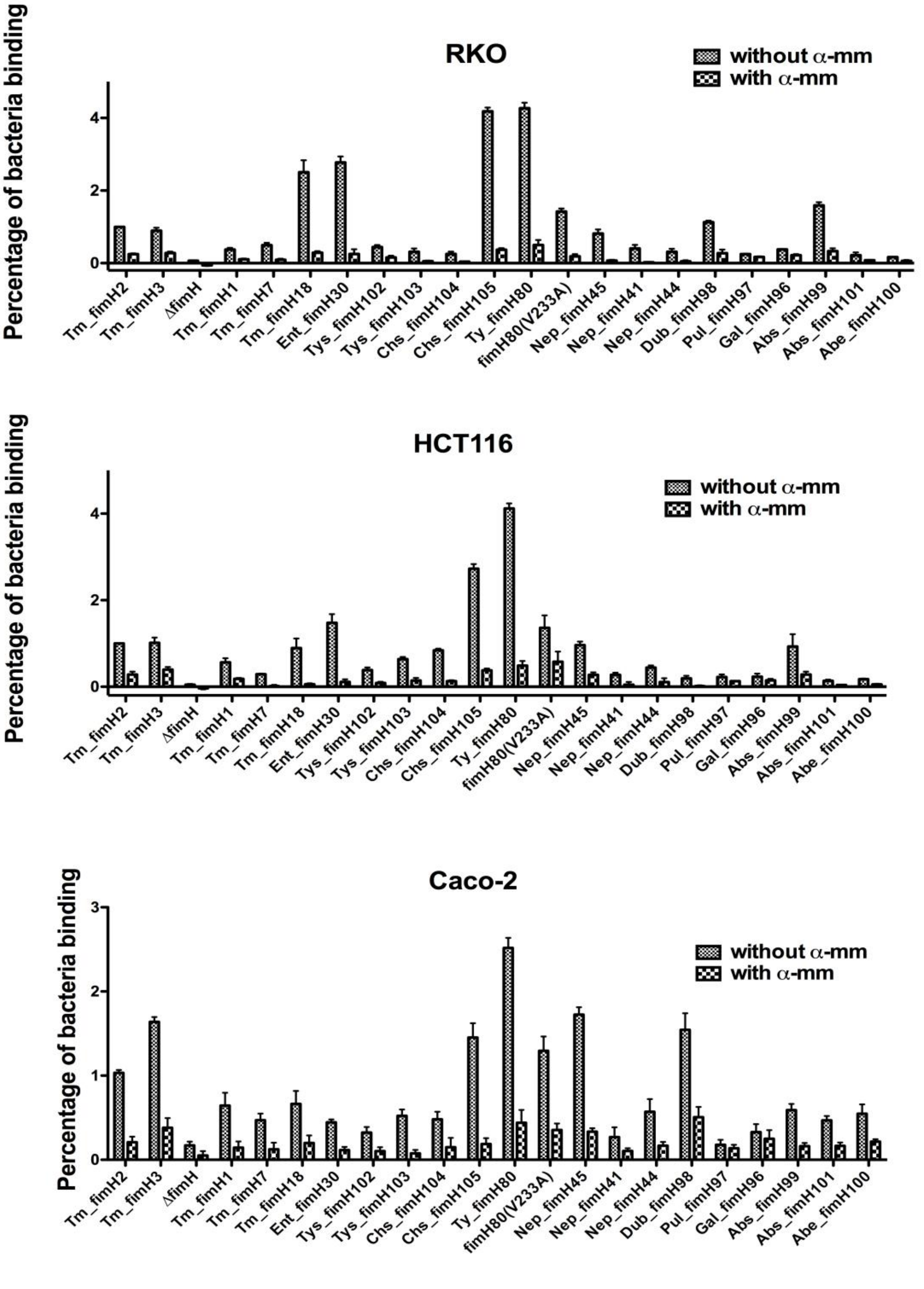

Supplementary Fig. 8. Bacterial adhesion to human intestinal epithelial cells. Binding of recombinant *E. coli* expressing *Salmonella* type 1 fimbriae with different FimH alleles, as listed in Supplementary Fig. 9. Three human intestinal epithelial cell lines (RKO, HCT116, Caco-2) were used for the binding assays. The data are averages with SEM from at least four replicated assays. All the binding assays were done in the presence or absence of methyl-D-mannopyranoside (α-mm).

Supplemental Figures

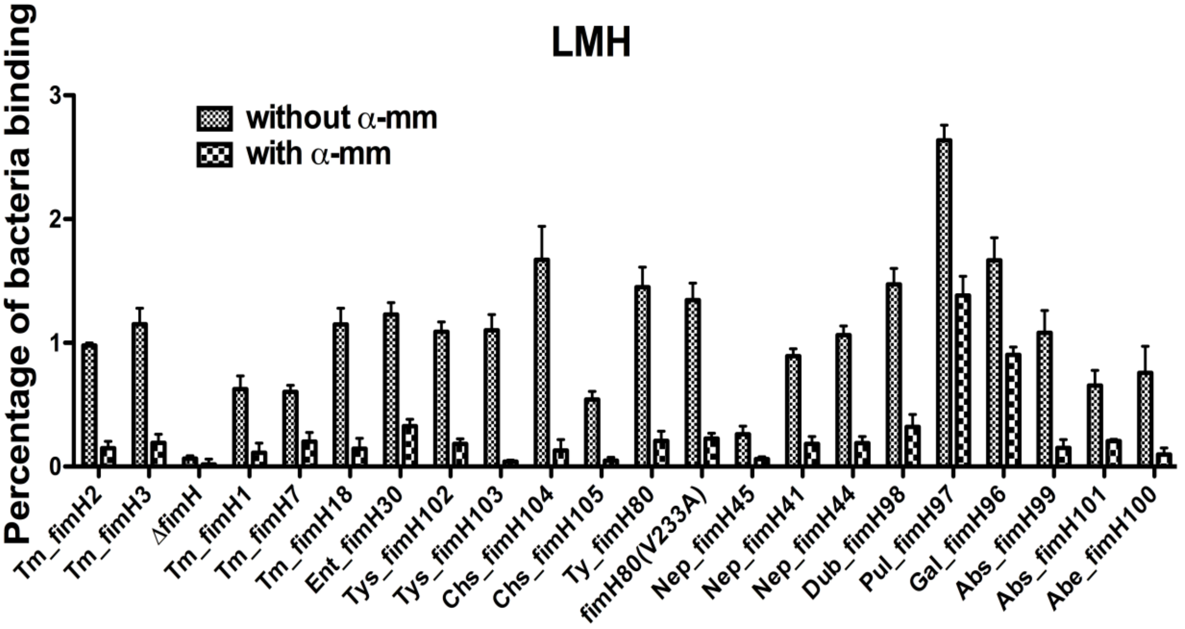

Supplementary Fig. 9. Bacterial adhesion to chicken liver epithelial cells. Binding of recombinant *E. coli* expressing *Salmonella* type 1 fimbriae with different FimH alleles, as listed in Supplementary Fig. 9. One chicken liver epithelial cell line (LMH) was used for the binding assays. The data are averages with SEM from at least four replicated assays. All the binding assays were done in the presence or absence of methyl-D-mannopyranoside (α-mm).

Supplemental Figures

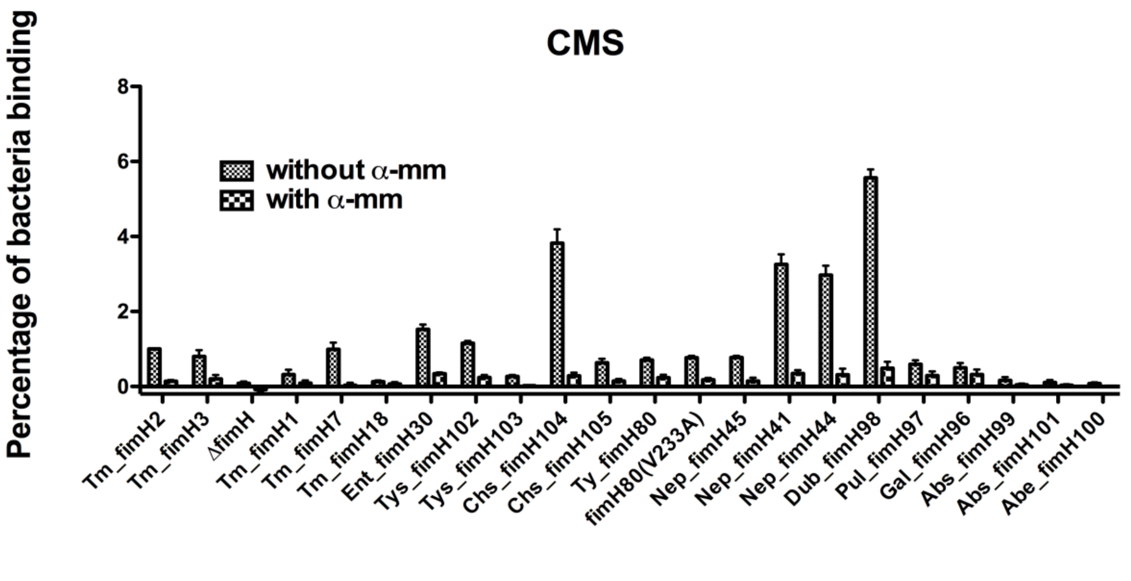

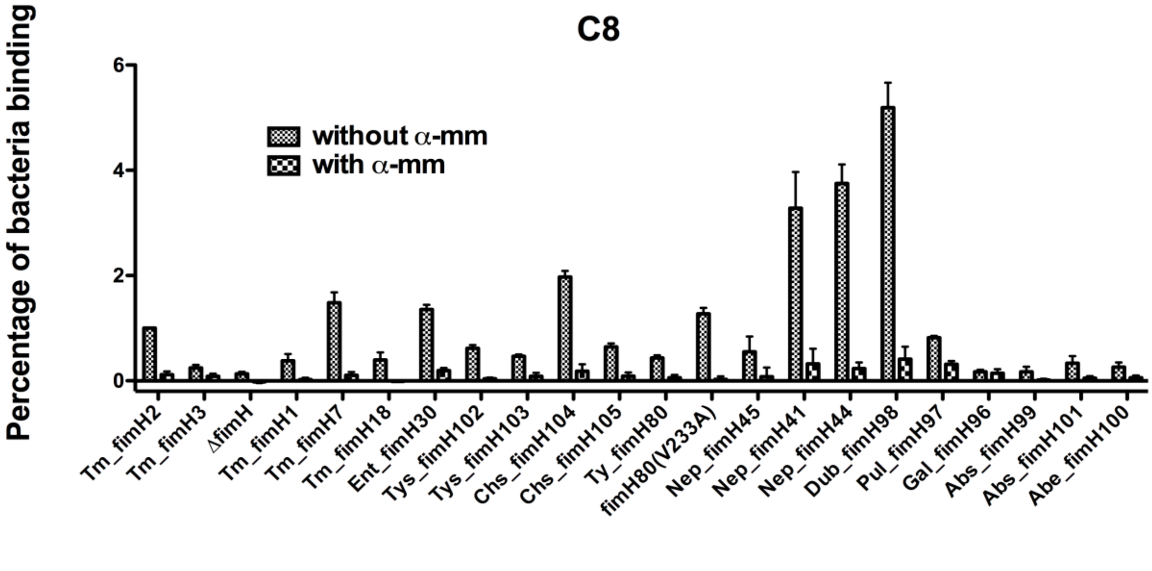

Supplementary Fig. 10. Bacterial adhesion to bovine intestinal epithelial cells. Binding of recombinant *E. coli* expressing *Salmonella* type 1 fimbriae with different FimH alleles, as listed in Supplementary Fig. 9. Two bovine intestinal epithelial cell lines (C8 and CMS) were used for the binding assays. The data are averages with SEM from at least four replicated assays. All the binding assays were done in the presence or absence of methyl-D-mannopyranoside (α-mm).

Supplemental Figures

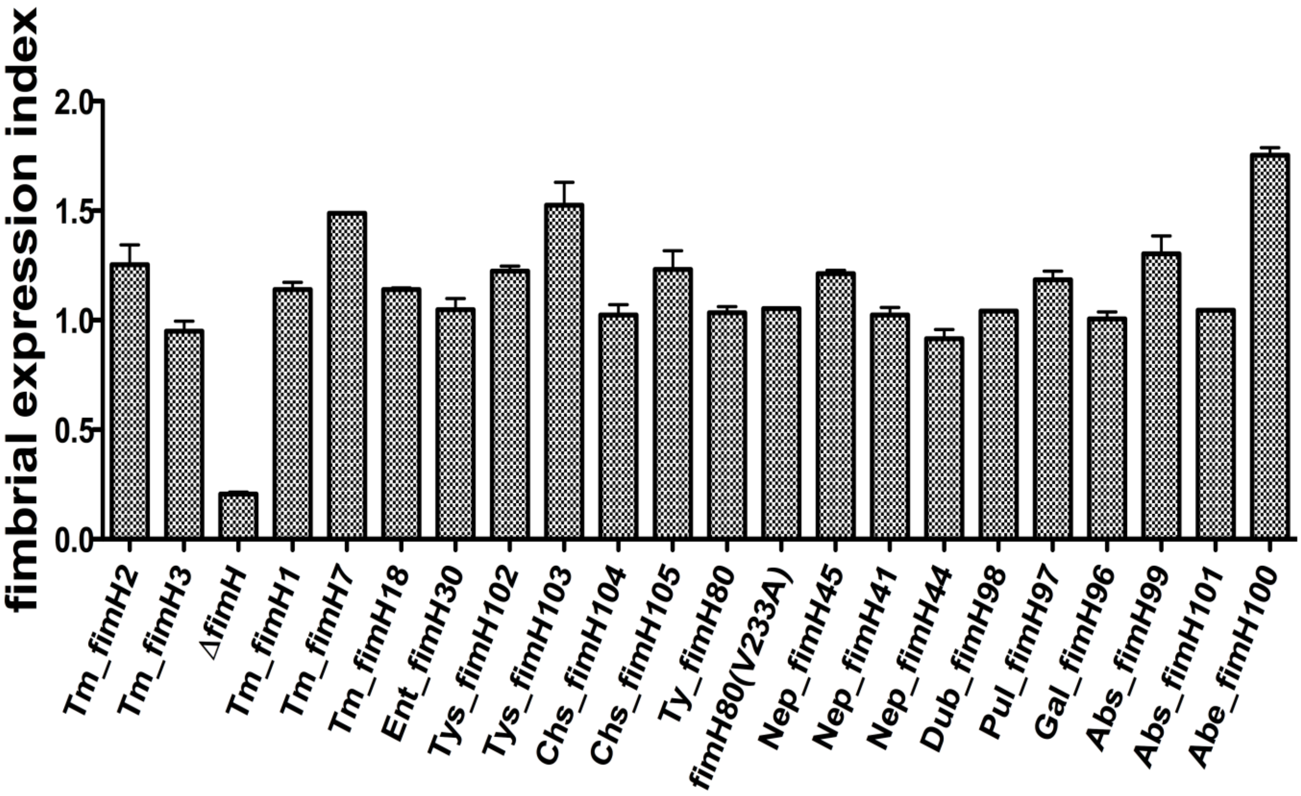

Supplementary Fig. 11. Expression level of recombinant *Salmonella* type 1 fimbriae with the different FimH allele. Fimbriae expression indices were calculated as described in Materials and Methods. The means and SEM are from one representative experiment of three repeated experiments.

Supplementary Tables

Supplementary Table 1. List of phage related genes that were shared among the *S.* Typhimurium strains.

Host specific gene loci	Host groups	Gene Function	Functional Category	Note
**SL1344_0286**	bovine	ClpB chaperone protein	Phages, Prophages, Transposable elements	Type VI SS
**SL1344_0988**	bovine	Phage tail fiber protein	Phages, Prophages, Transposable elements	Phage structure protein
**SL1344_2586**	bovine	hypothetical protein	Phages, Prophages, Transposable elements	Phage region unknown protein
**SL1344_2588**	bovine	putative methyltransferase	Phages, Prophages, Transposable elements	Phage region protein
**SL1344_2587**	bovine	Phage EaA protein	Phages, Prophages, Transposable elements	Phage region protein
**SL1344_2594**	bovine	ATPase domain protein	Phages, Prophages, Transposable elements	Phage region protein
**SL1344_2597**	bovine	Exodeoxyribonuclease VIII (EC 3.1.11.-)	Phages, Prophages, Transposable elements	Phage region protein
**SL1344_2589**	bovine	Phage EaD protein	Phages, Prophages, Transposable elements	Phage region protein
**SL1344_2590**	bovine	Origin specific replication binding factor	Phages, Prophages, Transposable elements	Phage region protein
**SL1344_2591**	bovine	Replication protein O	Phages, Prophages, Transposable elements	Phage region protein
**SL1344_2592**	bovine	putative DNA-binding protein	Phages, Prophages, Transposable elements	Phage region protein
**SL1344_2634**	bovine	hypothetical protein	Phages, Prophages, Transposable elements	Phage region protein
**SL1344_2690**	bovine	Phage terminase, endonuclease subunit	Phages, Prophages, Transposable elements	Phage region protein
**SL1344_2700**	bovine	hypothetical protein	Phages, Prophages, Transposable elements	Phage region protein
**UMN798_1786**	porcine	Putative inner membrane protein	Phages, Prophages, Transposable elements	Phage region protein
**STM14_1847**	chicken	hypothetical protein	Unknown	Unknown function
**STM14_2032**	chicken	Transposase IS200 like	Phages, Prophages, Transposable elements	Mobile element
**STM14_2427**	chicken	hypothetical protein	Phages, Prophages, Transposable elements	Phage region protein
**STM14_2429**	chicken	hypothetical protein	Phages, Prophages, Transposable elements	Phage region protein

Supplementary Tables

Supplementary Table 2. General Statistics of 15 surface genes of *Salmonella* Typhimurium

Gene[#]	No. of Seq.	Length of Seq.	Indel (bp)	SM[¶]	NSM[¶]	P[‡]	Tajima's D value[€]	Statistical significance	a[†]	A[†]
bcfD	580	1005	0	31	12	0.0011	-2.21842	**, P < 0.01	22	13
fimH	580	1008	3	32	28	0.00199	-2.14133	**, P < 0.01	29	26
lpfD	580	1077	10	-	-	0.00026	-2.62149	***, P < 0.001	29	-
***lpfD*_CDS**[Σ]	287	1077	0	13	14	0.0004	-2.46145	**, P < 0.01	21	13
***lpfD*_pseudo**	293	1067	0	-	-	0.00012	-2.39226	**, P < 0.01	11	-
ompA	580	1056	6	18	6	0.00037	-2.2222	**, P < 0.01	14	6
ompC	580	1140	6	43	18	0.00138	-2.45453	**, P < 0.01	31	20
ompN	580	1131	0	81	48	0.00107	-2.74782	***, P < 0.001	38	25
pefA	367	516	0	6	6	0.00015	-2.18578	**, P < 0.01	11	7
safD	580	468	12	15	17	0.00112	-2.31334	**, P < 0.01	20	16
stbD	580	1323	0	35	30	0.00092	-2.31334	**, P < 0.01	30	24
stcD	580	1005	6	8	7	0.00018	-2.10034	**, P < 0.01	12	8
stdD	580	1107	39	21	32	0.00085	-2.5133	***, P < 0.001	27	24
stfH	580	837	12	28	42	0.00074	-2.66028	***, P < 0.001	24	20
sthE	580	1083	0	37	19	0.0013	-2.28432	**, P < 0.01	24	18
stiH	580	1077	0	46	36	0.00073	-2.65528	***, P < 0.001	23	17
stjA	580	570	0	12	12	0.00043	-2.32935	**, P < 0.01	14	12

[#] None of the genes studied displayed a history of recombination, as tested with MaxChi (Piganeau, et al., 2004) or PhylPro (Weiller GF, 1998) at the 95% significance level, to exclude hotspot-like changes resulting from recombination and not mutation.

[¶] Synonymous mutations (SM) and nonsynonymous mutations (NSM)

[‡] Pi: Measurement of nucleotide diversity (Borowsky RL, 2001).

[€] Tajima's D value (Tajima F, 1989): Tests the null hypothesis of mutation-drift equilibrium and constant population size; statistical significance of an excess of common as compared to rare nucleotide variants, via the difference between two different estimates of nucleotide diversity. If the Tajima's D value is smaller than -2.0, P < 0.01 (**), if the value is smaller than -2.5, P < 0.001 (***).

[†] Numbers of nucleotide (a) and protein (A) alleles

[Σ] Full length gene (CDS, coding DNA sequence) or pseudogene (pseudo)

Supplementary Tables

Supplementary Table 3. Summary of 16 detected host-associated mutations in *S.* Typhimurium.

Gene	Nt Position	Mutation Type	Nt Substitution	Aa Substitution	Association (Nt)
bcfD	301	nsSNP	T/C	Ser/Pro	Avian (C)
	731	nsSNP	G/A, A/G	Gly/Asp, Asp/Gly	Human (A); Porcine (G)
	781	nsSNP	T/G	Phe/Val	Bovine (G)
fimH	737	nsSNP	T/C, C/T	Val/Ala, Ala/Val	Bovine (C); Human (T)
pefA	n.a.[a]	Absent gene	n.a.[a]	n.a.[a]	Bovine (Presence)
lpfD	399-408	Pseudogene	GTTTGAGAAT/-	n.a.[b]	Bovine (10bp deletion)
safD	170	nsSNP	T/C	Val/Ala	Avian (C)
stcD	700	nsSNP	G/A	Ala/Thr	Bovine (A)
stdD	932	nsSNP	G/A	Gly/Asp	Bovine (A)
stfH	418	nsSNP	G/A	Ala/Thr	Bovine (A)
	775	nsSNP	A/C	Ser/Arg	Bovine (C)
	790	nsSNP	A/G	Arg/Gly	Bovine (G)
sthE	887	nsSNP	A/G	Asp/Gly	Human (G)
stiH	628	nsSNP	G/A	Val/Met	Avian (A)
stjA	43	nsSNP	C/A	Leu/Ile	Bovine (A)
	517	nsSNP	T/G	Ser/Ala	Bovine (G)

[a] *pefA* is on a plasmid that is mainly present in bovine isolates

[b] A 10 bp deletion that results in a frameshift mutation

Supplementary Tables

Supplementary Table 4. DNA Polymorphism in different domains encoded by *fimH* among 580 *S.* Typhimurium isolates.

Domain	Sequence Range	Synonymous Mutation	Nonsynonymous Mutation	Pi*	Tajima's D Value#	Statistical Significance
Signal peptide	1-66	1	0	0.00046	-0.6852	n.s.
Lectin domain	67-588	17	19	0.00209	-2.0963	**, P < 0.01
Linker	589-597	0	0	0	n.a¶	n.a.
Pilin domain	598-1008	14	9	0.00215	-1.8255	n.s.
Full gene	1-1008	32	28	0.00199	-2.1413	**, P < 0.01

* Pi (π) measures nucleotide diversity (Borowsky RL, 2001).
# Tajima's D value (Tajima F, 1989): Tests the null hypothesis of mutation-drift equilibrium and constant population size; statistical significance of an excess of common as compared to rare nucleotide variants, via the difference between two different estimates of nucleotide diversity. If the Tajima's D value is smaller than -2.0, P < 0.01 (**). Values between -2 and +2 are considered not significant (n.s.).
¶ n.a.: not available.

Supplementary Tables

Supplementary Table 5. DNA Polymorphism in different domains encoded by *fimH* among 1848 *S.* enterica subspecies I isolates.

Domain	Sequence Range	Synonymous Mutation	Nonsynonymous Mutation	Pi*	Tajima's D Value#	Statistical Significance
Signal peptide	1-66	5	8	0.0054	-2.1337	**, P < 0.01
Lectin domain	67-588	37	48	0.176	-2.7385	***, P < 0.001
Linker	589-597	1	0	0.00012	-0.7597	n.s.
Pilin domain	598-1011	29	30	0.01239	-1.7833	n.s.
Full gene	1-1011	72	86	0.01223	-2.1628	**, P < 0.01

* Pi (π) measures nucleotide diversity (Borowsky RL, 2001).
# Tajima's D value (Tajima F, 1989): Tests the null hypothesis of mutation-drift equilibrium and constant population size; statistical significance of an excess of common as compared to rare nucleotide variants, via the difference between two different estimates of nucleotide diversity. If the Tajima's D value smaller than -2.0, P < 0.01 (**), or smaller than -2.5, P < 0.001 (***). Values between -2 and +2 are considered not significant (n.s.).

Supplementary Tables

Supplementary Table 6. Primers used for targeted parallel sequencing.

Genes	Tag name	Oligonucleotide Sequence
stcD	stcD-F1	ACACTGACGACATGGTTCTACAgtcggtagtggtgaggtggt
	stcD-F2	ACACTGACGACATGGTTCTACATCCGATATCATCGTGGGATT
	stcD-F3	ACACTGACGACATGGTTCTACAAAGCGCTTCTTCTGCAACTC
	stcD-F4	ACACTGACGACATGGTTCTACATACGATCGCAAGCAGACAAC
	stcD-R1	TACGGTAGCAGAGACTTGGTCTGGCCGTAAACTGAGTTGCAG
	stcD-R2	TACGGTAGCAGAGACTTGGTCTGCGTGATCGTCAGATCTTCA
	stcD-R3	TACGGTAGCAGAGACTTGGTCTGAAGCATTACCCTGCTCTGC
	stcD-R4	TACGGTAGCAGAGACTTGGTCTCACATAGATCAGCGCAGTCG
safD	safD-F1	ACACTGACGACATGGTTCTACAatcaaatgagcggtctctgc
	safD-F2	ACACTGACGACATGGTTCTACAAGTAGCTTGGTGCCGATAGC
	safD-R1	TACGGTAGCAGAGACTTGGTCTCCACACTGAAACTGGCGTTA
	safD-R2	TACGGTAGCAGAGACTTGGTCTgacggCTACGTATCCTCCTG
bcfD	bcfD-F1	ACACTGACGACATGGTTCTACAtctctcttcctcccgtcaga
	bcfD-F2	ACACTGACGACATGGTTCTACAGCCATATCCGGTCGTAGAAA
	bcfD-F3	ACACTGACGACATGGTTCTACAGCAAAGCTGTGAAATCAACG
	bcfD-R1	TACGGTAGCAGAGACTTGGTCTTCCCATCAGGACATTGTTCA
	bcfD-R2	TACGGTAGCAGAGACTTGGTCTGGTTGAAATTGCCGCTGTAT
	bcfD-R3	TACGGTAGCAGAGACTTGGTCTgcgcagtgtgatctgttcat
fimH	fimH-F1	ACACTGACGACATGGTTCTACActgaagcaggcgattacgat
	fimH-F2	ACACTGACGACATGGTTCTACAGGGACAACGGTGAATTATACCT
	fimH-F3	ACACTGACGACATGGTTCTACAGCCAGACAATGTTTACCGTCT
	fimH-F4	ACACTGACGACATGGTTCTACAGGTCGTGGAGTTTGATTTCG
	fimH-R1	TACGGTAGCAGAGACTTGGTCTCAGCGACACTATCGGTGATG
	fimH-R2	TACGGTAGCAGAGACTTGGTCTTATCGCCGAAATCAAACTCC
	fimH-R3	TACGGTAGCAGAGACTTGGTCTGGCGGCGTTATCATCAAG
	fimH-R4	TACGGTAGCAGAGACTTGGTCTcacaaccgatagcgatgaaa
stbD	stbD-F1	ACACTGACGACATGGTTCTACAccaactcgatcttcacacca
	stbD-F2	ACACTGACGACATGGTTCTACAGCACGCCCTATTCCAGTAAA
	stbD-F3	ACACTGACGACATGGTTCTACACCAATGGACCGAGATACCTG
	stbD-F4	ACACTGACGACATGGTTCTACACGTCAGCCTGGAATGTGAG
	stbD-R1	TACGGTAGCAGAGACTTGGTCTCGCCATTTGTCGAATACATCT
	stbD-R2	TACGGTAGCAGAGACTTGGTCTAATAATCGCTGGCATGATCC
	stbD-R3	TACGGTAGCAGAGACTTGGTCTTGTCCAGTAGCCACGACAGA
	stbD-R4	TACGGTAGCAGAGACTTGGTCTATTGCGCATTCACGCTTC
sthE	sthE-F1	ACACTGACGACATGGTTCTACAcgttgtgacgatcactattgc
	sthE-F2	ACACTGACGACATGGTTCTACAGACAATCAATGCGAATGACG
	sthE-F3	ACACTGACGACATGGTTCTACATGCCATCACCGTGGATTTA
	sthE-R1	TACGGTAGCAGAGACTTGGTCTTATCGTAAGGGCCAGCATTC
	sthE-R2	TACGGTAGCAGAGACTTGGTCTGGATATCCTGCTCGCCTGT
	sthE-R3	TACGGTAGCAGAGACTTGGTCTTTAATGTTGCCGTGGCATT
stdD	stdD-F1	ACACTGACGACATGGTTCTACAggactgcagatgaatcgctac
	stdD-F2	ACACTGACGACATGGTTCTACACTAGATGCGATGGGACTTGG
	stdD-F3	ACACTGACGACATGGTTCTACATCTCAGAAACGGATTGGGTAA
	stdD-R1	TACGGTAGCAGAGACTTGGTCTTCCCAGGAGAACGAAACAAC
	stdD-R2	TACGGTAGCAGAGACTTGGTCTTGCCTGTCTGGTCTGTGAAG
	stdD-R3	TACGGTAGCAGAGACTTGGTCTTAACGGGTATTGCAGCAGAA
stiH	stiH-F1	ACACTGACGACATGGTTCTACAgtctgccagtgaggaacaca

Supplementary Tables

	stiH-F2	ACACTGACGACATGGTTCTACAGACCGGCGATTTAACACAAA
	stiH-F3	ACACTGACGACATGGTTCTACACCATTCCTGAGACGACGATT
	stiH-F4	ACACTGACGACATGGTTCTACAGCACGTCACCACTCAGGTAA
	stiH-R1	TACGGTAGCAGAGACTTGGTCTCGATGTAGGTTTCCGTACCAA
	stiH-R2	TACGGTAGCAGAGACTTGGTCTCGGTAGCCTGACCTCGATAA
	stiH-R3	TACGGTAGCAGAGACTTGGTCTGCTGCCTCTACCGTCAGTGT
	stiH-R4	TACGGTAGCAGAGACTTGGTCTgttgcgcttcgcttcact
stfH	stfH-F1	ACACTGACGACATGGTTCTACAgggaaggttctctgcaagtg
	stfH-F2	ACACTGACGACATGGTTCTACAGCCGAAGGTTGTATTGAAGG
	stfH-F3	ACACTGACGACATGGTTCTACAGATATCTCCTGGGATGCGAAC
	stfH-R1	TACGGTAGCAGAGACTTGGTCTGCAACCATAATCATCGCTTTG
	stfH-R2	TACGGTAGCAGAGACTTGGTCTTTGAGCGTAATACCCGATGA
	stfH-R3	TACGGTAGCAGAGACTTGGTCTcgtccagaaacaacgtaggc
lpfD	lpfD-F1	ACACTGACGACATGGTTCTACAgataataccggcgtggtgat
	lpfD-F2	ACACTGACGACATGGTTCTACACCTATGCGATGTCCTGTGAA
	lpfD-F3	ACACTGACGACATGGTTCTACAATCCGTTTGTGGGTGAAAGT
	lpfD-F4	ACACTGACGACATGGTTCTACAAAGAACGCCACGAAATTCAC
	lpfD-R1	TACGGTAGCAGAGACTTGGTCTTTGATCTGGTAATAGGAGCGACT
	lpfD-R2	TACGGTAGCAGAGACTTGGTCTGCCCTGAGGTACCGTAATTG
	lpfD-R3	TACGGTAGCAGAGACTTGGTCTACAAGACCCGATACGCTCAG
	lpfD-R4	TACGGTAGCAGAGACTTGGTCTcggaagcggtaagcagtaaa
stjA	stjA-F1	ACACTGACGACATGGTTCTACAtatgaaacggcaaagggagt
	stjA-F2	ACACTGACGACATGGTTCTACATTGCCCAGAAAGCCAGAATA
	stjA-R1	TACGGTAGCAGAGACTTGGTCTGGGTGCTGGCAATAGGATAA
	stjA-R2	TACGGTAGCAGAGACTTGGTCTatcgcactggaatccgtact
pefA	pefA_F1	ACACTGACGACATGGTTCTACAcctgtccgggaaggaataac
	pefA_F2	ACACTGACGACATGGTTCTACATTGTTGACTTCGCCATGAAA
	pefA_R1	TACGGTAGCAGAGACTTGGTCTCAGAAGCCCAGGAAACAGTG
	pefA_R2	TACGGTAGCAGAGACTTGGTCTgcctgcaatcagcgatataaa
ompA	ompA_F1	ACACTGACGACATGGTTCTACAgatgataacgaggcgcaaa
	ompA_F2	ACACTGACGACATGGTTCTACACCGTCTGGGTGGTATGGTAT
	ompA_F3	ACACTGACGACATGGTTCTACAATCTACCCTGAAGCCGGAAG
	ompA_R1	TACGGTAGCAGAGACTTGGTCTCCGGTGTCGTGGTCTTTAGT
	ompA_R2	TACGGTAGCAGAGACTTGGTCTGTGAAGCCCAGAACGACAAC
	ompA_R3	TACGGTAGCAGAGACTTGGTCTaaaggcgttgtcatccagac
ompC	ompC_F1	ACACTGACGACATGGTTCTACAtgccgactggttaatgagg
	ompC_F2	ACACTGACGACATGGTTCTACAAAATTCGCTGATGCAGGTTC
	ompC_F3	ACACTGACGACATGGTTCTACAACGCTCGCCTGTATGGTAAC
	ompC_R1	TACGGTAGCAGAGACTTGGTCTCAGGAGGTCACGTCATAGGTT
	ompC_R2	TACGGTAGCAGAGACTTGGTCTTGTTCGCATCGTATTTCAGG
	ompC_R3	TACGGTAGCAGAGACTTGGTCTCTACGATGTCGTCGGTGTTG
ompN	ompN_F1	ACACTGACGACATGGTTCTACAgcctttgattcaacgaatctg
	ompN_F2	ACACTGACGACATGGTTCTACACTGAAATTCGGCGACTACG
	ompN_F3	ACACTGACGACATGGTTCTACATCGTAACGTGAAGAACGCTAAC
	ompN_F4	ACACTGACGACATGGTTCTACAATGGACTGTTGGCGCTAAAT
	ompN_R1	TACGGTAGCAGAGACTTGGTCTATCGGTCCAGCCTTCTACG
	ompN_R2	TACGGTAGCAGAGACTTGGTCTCCGCCAGCGGTAGAGTAAT
	ompN_R3	TACGGTAGCAGAGACTTGGTCTCAGGTCTTTATCATCGCCATT
	ompN_R4	TACGGTAGCAGAGACTTGGTCTggcgggcttgagaagaat

Supplementary References

1. Piganeau G, Gardner M, Eyre-Walker A. A broad survey of recombination in animal mitochondria. *Mol Biol Evol* **21**, 2319-2325 (2004).

2. Weiller GF. Phylogenetic profiles: a graphical method for detecting genetic recombinations in homologous sequences. *Mol Biol Evol* **15**, 326-335 (1998).

3. Borowsky RL. Estimating nucleotide diversity from random amplified polymorphic DNA and amplified fragment length polymorphism data. *Molecular phylogenetics and evolution* **18**, 143-148 (2001).

4. Tajima F. Statistical method for testing the neutral mutation hypothesis by DNA polymorphism. *Genetics* **123**, 585-595 (1989).
